# Low reporting quality limits the contribution of human organ models to COVID-19 research: a systematic review

**DOI:** 10.1016/j.ebiom.2026.106392

**Published:** 2026-07-28

**Authors:** Maren Hülsemann, Silke Kniffert, Anna Löwa, Morris Baumgardt, Katja Hönzke, Valeria Fernandez Vallone, Natascha I. Drude, Thomas Stüdemann, Ahmed S.M. Ali, Maria Arroyo Araujo, Alexandra Bannach-Brown, Linda Brunotte, Aileen Faist, Diana Fatykhova, Mara Fischer, Lina Hellwig, Saskia Hinse, Mirjana Kessler, Benjamin G. Carlisle, Sadaf Khankeh, Ishminder Singh Dhamrait, Charlotte Klein, Hristina Koceva, Sriram Kumar, Jens Kurreck, Katarzyna A. Ludwik, Philipp Mergenthaler, Sarah S. Schmerbeck, Sameer Singh, Luiz Gustavo Teixeira Alves, Emanuel Wyler, Harald Stachelscheid, Stefan Hippenstiel, Ulf Toelch, Andreas C. Hocke

**Affiliations:** a Berlin Institute of Health at Charité – Universitätsmedizin Berlin, QUEST Center for Responsible Research, Berlin, Germany; b Department of Infectious Diseases, Respiratory Medicine and Critical Care Medicine, Charité – University Medical Center Berlin, Corporate Member of Freie Universität Berlin and Humboldt-Universität zu Berlin, Germany; c Berlin Institute of Health at Charité—Universitätsmedizin Berlin, Core Unit Pluripotent Stem Cells and Organoids (CUSCO), Berlin, Germany; d Berlin Institute for Medical Systems Biology, Max Delbrueck Center in the Helmholtz Association, Berlin, Germany; e Technische Universität Berlin, Department of Applied Biochemistry, Berlin, Germany; f Institute of Virology, University Hospital Münster, University of Münster, Germany; g Center for Stroke Research Berlin, Charité – University Medical Center Berlin, Corporate Member of Freie Universität Berlin and Humboldt-Universität zu Berlin, Berlin, Germany; h Department of Obstetrics and Gynecology, Ludwig-Maximilians-Universität München, Munich, Germany; i Center for Sepsis Control and Care, Institute of Biochemistry II, Universitätsklinikum Jena, Germany; j Institute of Medical Physics and Biophysics, Charité – University Medical Center Berlin, Corporate Member of Freie Universität Berlin and Humboldt-Universität zu Berlin, Berlin, Germany; k Department of Neurology with Experimental Neurology, Charité – University Medical Center Berlin, Corporate Member of Freie Universität Berlin and Humboldt-Universität zu Berlin, Berlin, Germany; l Radcliffe Department of Medicine, University of Oxford, Oxford, UK; m Institute of Social and Preventative Medicine, University of Bern, Switzerland; n Immunology of Respiratory Tract Infections, Medical Clinic V, University Giessen, Germany; o Brandenburg Technische Universität Cottbus-Senftenberg, Faculty of Health Sciences, Senftenberg, Germany; p Department of Anesthesiology, Intensive Care, and Pain Medicine, University Hospital Muenster, Germany

**Keywords:** COVID-19, Human organ models, Organoids, Systematic review, Reporting quality, Meta-analysis

## Abstract

**Background:**

The COVID-19 pandemic created a unique situation in which researchers repurposed human models of varying complexity to investigate SARS-CoV-2, providing the opportunity to analyse their contribution across organs.

**Methods:**

We conducted a systematic review of 558 studies, divided into pandemic and post-pandemic periods, to assess how human models were applied to investigate host factors, viral replication of SARS-CoV-2, immune responses, and to evaluate reporting quality.

**Findings:**

Our analysis revealed substantial limitations that were only partially alleviated in post-pandemic studies. These limitations included heterogeneity of outcome measures, incomplete documentation of model characteristics and experimental procedures, and variable reporting quality, which were associated with reduced cross-study comparability, more difficult risk-of-bias assessment, and limited reliability and interpretability of evidence synthesis and meta-analytical results.

**Interpretation:**

Strengthening community-driven reporting standards and methodological transparency is essential to enable robust evidence synthesis and to fully realise the potential of human organ models in future pandemics and translational research.

**Funding:**

The study was supported by 10.13039/501100006188Einstein Foundation Berlin; 10.13039/501100002347Federal Ministry of Research, Technology and Space; 10.13039/501100000780European Union; 10.13039/501100003042Else Kröner-Fresenius-Stiftung; 10.13039/501100001663Volkswagen Foundation; Charité 3R; Foundation Charité.

**Research in context**

**Evidence before this study**

To conduct this study, we searched PubMed and the WHO COVID-19 database for articles published from December 2019 to February 2022 (pandemic period) as well as from March 2022 to July 2025 (post-pandemic period), without restricting to English language publications. We aimed to identify primary research using human primary cell models, organoids, or tissue cultures to study SARS-CoV-2. Our search strategy combined key terms related to the disease, model systems, host factors, and organisms. Studies were included if they described experimental work using human *in vitro* or *ex vivo* models to investigate SARS-CoV-2 host factors, viral replication, or immune responses. Out of 2220 identified studies, 558 met our eligibility criteria. A comprehensive data extraction covering model generation, experimental design, and experimental results was performed. Risk of bias was indirectly assessed through detailed quality-of-reporting evaluations. Although valuable findings were reported in individual studies, the overall quality and consistency of reporting were highly variable, hampering reliable synthesis of evidence. Meta-analyses were attempted on subsets of data but were limited by reporting deficiencies and study heterogeneity.

**Added value of this study**

This study comprehensively evaluates the contribution of human organ models to understanding COVID-19 across multiple organ systems and model types. Unlike previous reviews that focused on single organs or technical aspects, we provide an integrated overview of how human models were applied during the COVID-19 pandemic and assess their potential for evidence synthesis. Our analysis reveals significant shortcomings in reporting practices, which limit the interpretability and comparability of findings across studies. We show that while human organ models have generated important insights into SARS-CoV-2 biology, their broader impact is curtailed by inconsistent documentation of model characteristics, experimental procedures, and metadata. Our work highlights the urgent need for improved standardisation and transparency in human model research.

**Implications of all the available evidence**

The available evidence underscores the promise of human organ models in infectious disease research but also reveals major obstacles that must be overcome to fully harness their potential. Clear and consistent reporting standards are essential to enable evidence synthesis, comparative assessments, and knowledge transfer across studies. Improving methodological transparency will strengthen the reliability of human organ models as translational tools, support their integration into biomedical research pipelines, and enhance their contribution to pandemic preparedness and clinical innovation. Future research should prioritise detailed documentation of model generation, experimental design, and sample characteristics to ensure that human models can serve as robust foundations for scientific discovery and public health decision-making.

## Introduction

### Background and rationale

Human organ models comprise *in vitro* systems using primary or stem cells to form either a two-dimensional (2D) layer or complex 3D structures, such as air-liquid interface (ALI) or organoid models. Human organ models also include tissue samples cultivated *ex vivo* from surgical material, biopsies, or autopsies. Overall, human organ models are advanced model systems applied in basic and preclinical research to close the gap from the classical 2D cell lines and animal models to the actual human organ.^1^

During health crises such as pandemics, the adherence to rigorous standards is crucial to minimise uncertainty and generate robust evidence for health care policy and clinical decision-making.^2^ With the onset of the COVID-19 pandemic, a modelling dilemma became evident, as it was largely unclear which species would be susceptible to SARS-CoV-2 and to what extent results obtained in other species had significance for humans.^3^ Animal models were initially not available to study the course of disease or to identify anti-viral drugs. Therefore, human organ models played a key role in addressing urgent research questions regarding SARS-CoV-2 pathogenesis and therapeutic treatment options.^4^^,^^5^

Human organ models have been developed to study disease mechanisms in various organs, including the liver, pancreas, skin, lungs, heart, and central nervous system. However, their potential contribution to the systemic investigation of multi-organ diseases remains unclear. The application of diverse models, each with distinct technical and biological characteristics, to a single disease represents a broad and heterogeneous approach in biomedical research. Undoubtedly, individual studies have made important contributions to our understanding of COVID-19 through these models.^5^ However, it is uncertain how well the results allow for evidence synthesis on key aspects such as virus replication, cellular tropism, host factor expression, or immune responses in individual tissue compartments. Additionally, it remains unclear whether synthesising this evidence can meaningfully advance our broader understanding of SARS-CoV-2. Specifically, can existing literature offer reproducible, valid, and translationally relevant findings? Answering these questions requires a systematic evaluation focused on quality. A structured synthesis of evidence combined with comparative analysis is essential for building a solid foundation to advance our understanding of diseases.

Systematic reviews (SRs) allow for such systematic categorisation and evaluation, identifying important trends, gaps and inconsistencies in literature. Initially conceived for clinical and recently also in vivo studies, SRs of *in vitro* studies now constitute an important and steadily growing contribution to the assessment of evidence for efficacy and toxicity/safety, or diverse methodological techniques. Current SRs on *in vitro* model systems focus on their predictive value or technical details in model generation. Across these SRs a lack of standardisation, quality criteria, common nomenclature and reporting standards is described for the literature applying *in vitro* models.^6^, ^7^, ^8^, ^9^, ^10^, ^11^

This SR spans across the whole research field of human organ models, investigating their contribution to the understanding of SARS-CoV-2 pathology, encompassing multiple organs, all published model types, and key methodologies to capture the diversity of approaches and reflect the comprehensiveness of research on COVID-19. A key focus of this SR is the quality of reporting and the suitability of experimental results for generating meaningful evidence summaries. Meaningful refers to results that are comparable across studies, with differences attributable to variations between the studies. Consequently, this comprehensive SR analyses currently available human organ models addressing comparable research questions within a shared disease context. The COVID-19 pandemic provides an extensive and diverse data set to examine the state of human organ model applications.

### Research focus

We developed four research questions to evaluate studies across different model types and organ systems. The first three were preregistered, while the fourth was added to assess the quality of reporting for experimental procedures and results. Based on this, we conducted a meta-analysis on a subset of the data.

1. What is the expression profile of SARS-CoV-2-relevant host factors in different human organ models?
2. In which organ and tissue compartments does SARS-CoV-2 replicate?
3. Which immune responses can be identified in the different SARS-CoV-2 infection models?
4. Does the quality of reporting on model generation, experimental procedures and results enable a clear understanding of the presented work and allow for a comparison across studies by meta-analysis?

## Methods

This SR was designed and performed in accordance with the Preferred Reporting Items for Systematic Reviews and Meta-Analyses (PRISMA guidelines, see Supplementary method 7 for details).^12^^,^^13^ A detailed description of the methods can be found in the supplementary information. Ethical approval was not required, and the statistical analysis is described in detail in the meta-analyses section.

### Search strategy and selection criteria

**Fig. 1:**
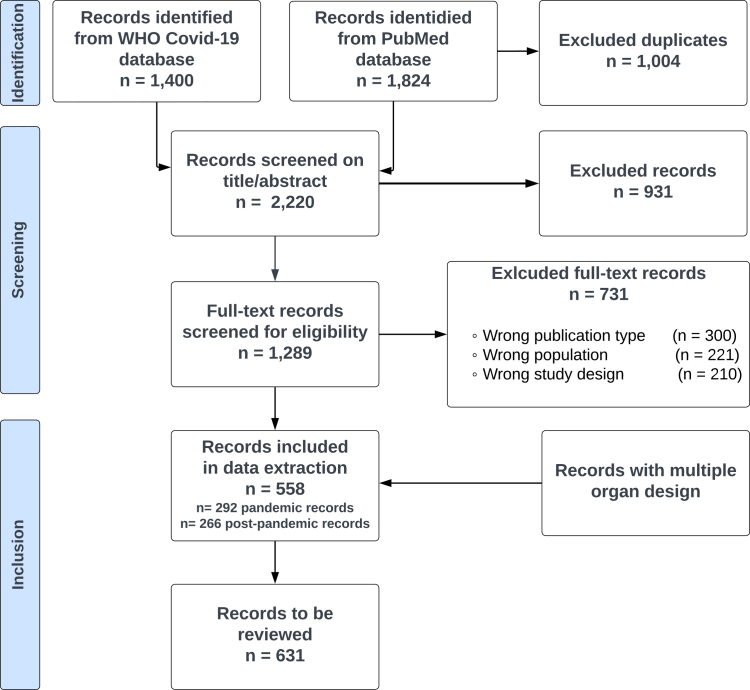
**PRISMA flow diagram for Systematic Review presenting the selection process of the included studies.** 3224 publications were identified from two databases. From these, 1004 duplicates were excluded, and 2220 records were screened on title and abstract, resulting in the exclusion of 931 records not meeting predefined eligibility criteria. Subsequently, 1289 full-text records were screened, of which 731 were excluded for reasons as described. In total, 292 records for the pandemic period, as well as 266 records for post-pandemic period were included for data extraction. Overall, 631 reviews had to be performed due to multiple organ foci in individual studies.

We grouped our research questions into key concepts to define the search terms (Supplementary Table 2). The systematic literature search was performed on PubMed and the WHO COVID-19 research database for the pandemic data set and on PubMed for the post-pandemic data set (WHO database was discontinued). Our search strategy identified 2220 studies published up to July 18, 2025 (Fig. 1) (see Supplementary method 4 and eligibility criteria Supplementary Table 3 for details). This SR was preregistered on the International Prospective Register of Systematic Reviews (PROSPERO, CRD42021250999) on June 9, 2021. Reporting quality questions were based on the MDAR checklist and refined with input from experts in human organ models, virology and RNA sequencing.^14^^,^^15^ (see Supplementary method 3 for details). Title and abstract screen was performed on RAYYAN to exclude non-primary research articles.^16^

#### Software

For data extraction Numbat Systematic Review Manager was used (RRID:SCR_019207).^17^ Data point extraction was performed in PlotDigitizer and recorded in Microsoft Excel.^18^ Meta-analyses and associated figures were conducted in R (RRID:SCR_001905).

### Validation of data extraction protocol

The data extraction protocol was piloted by five trained reviewers, and feedback from discussions led to refined questions and expanded explanations, resulting in a final question catalogue of 184 questions (104 questions on reporting quality and experimental design, 80 on data point extractions). For the post-pandemic data set we streamlined the question catalogue to 23 questions, based on the main outcomes of the analysis of the pandemic data.^15^

### Training

All 30 reviewers were initially inexperienced in SR methodology and completed an eight-week training (27 sessions), involving screening of ten studies, with continuous feedback, leading to a high level of consensus. Data points were extracted from plots by a specially trained team member using PlotDigitizer (see Meta-Analyses and Supplementary method 5 for details).

### Review process

Some of the 558 included studies used multiple models representing different organs; these studies were screened multiple times according to the organ focus. In total, this resulted in 631 individual reviews, with each expert reviewing between 28 and 49 studies. Each study in the pandemic data set (n = 292) was assessed by two independent reviewers from the 30-member team. In the case of discrepancies (∼60% of all studies) a third reviewer reconciled the assessment and provided the final answer. For the review of the post-pandemic data set (n = 266), the data extraction was streamlined on key questions addressed by 13 experienced reviewers (two per study). When clarification was needed, the reviewer directly coordinated with the co-reviewer to provide the final answer and ensure time efficiency (see Supplementary method 6 for details).

### Data collection, data handling and analysis

In the data extraction forms, answer options varied between single and multiple choice, free text and table response formats (see Supplementary method 6 for details). As there is no standardised terminology for outcome reporting of *in vitro* experiments (e.g., list of different y-axes descriptions as shown in Supplementary Tables 4 and 5), the reviewer training was time intensive (see Supplementary method 5 for details), and the data cleaning required manual manipulation. Data points were extracted from manuscript plots using PlotDigitizer, independently verified by two reviewers, and then reviewed by a statistician prior to meta-analysis. For descriptive analysis, Microsoft Excel and R were used. Data visualisations were performed with Origin.^19^

### Meta-analyses

#### Meta-analyses on host factor expression in respiratory tract models

In these meta-analyses, we compared qPCR-based expression of the key SARS-CoV-2 host factors ACE2 and TMPRSS2 across respiratory tract model types (cell models, organoids, and tissues), distinguishing between the proximal respiratory tract (pRT; nasal, tracheal, and bronchial epithelium) and the distal respiratory tract (dRT; primarily alveolar compartment) (see Supplementary method 1 for details).

#### Meta-analysis on viral replication in respiratory tract models

In this meta-analysis of qPCR-based SARS-CoV-2 replication in respiratory tract models, we applied a Bayesian non-linear hierarchical Gompertz growth model. Only experiments with ≥3 time points (hpi) were included, and replication values were z-transformed within each study to enable cross-study comparison. The final dataset comprised 130 data points from 13 studies (21 figures). Study heterogeneity was addressed by allowing replication rates to vary between studies, with effects estimated from posterior parameter distributions (see Supplementary method 2 for details).

### Data accessibility

Adjustments to our study protocol are reported in the preregistration.^15^ The search strategy, data extraction protocol, libraries of all studies separated by organ groups, coding of the meta-analyses and the GSE numbers of the RNA sequencing data sets are made available on OSF under a CC-BY licence.^15^

### Role of funders

The funders had no influence on the study design, data collection, analysis, or interpretation of the data, nor on the writing or submission of the manuscript.

## Results

### Overview of the model systems

**Fig. 2:**
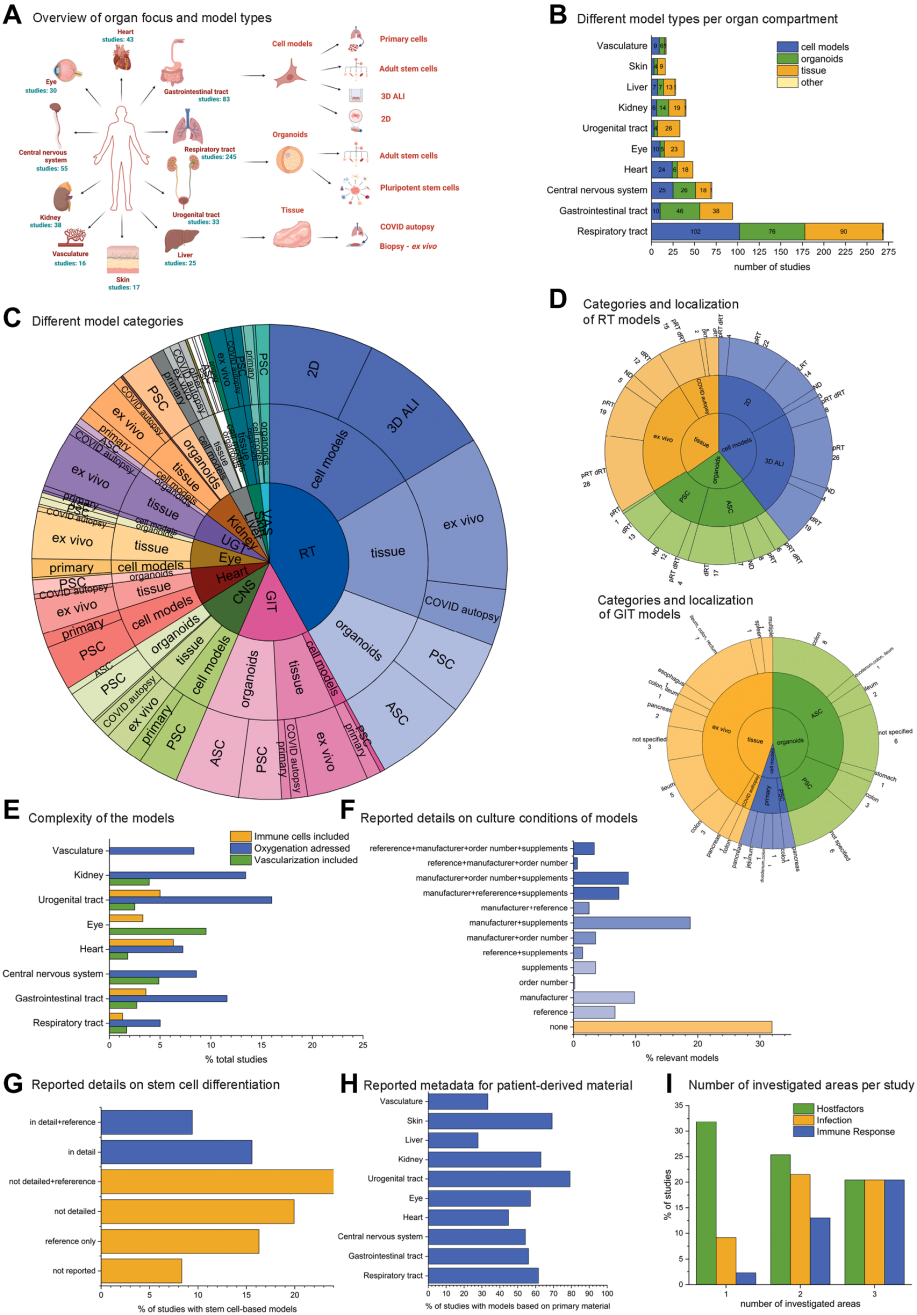
**Included human organ models categorised according to their generations, specifications and applications using pandemic and post-pandemic data. A.** Overview of organ groups represented by the investigated human models. The number of studies describing each organ system was extracted. The focus of our study was on primary cell- or stem cell-based models, organoids and tissue models further specified by different source materials and model details (created with BioRender.com). **B.** Number of models (cell models, organoids, tissue and other (OoC or bioprinted models)) per organ compartment. **C.** Further categorisation of each model based on the type of source material and its specific localisation within the respective organ, as reported in the studies (Supplementary Table 8). **D.** Detailed localisation descriptions for RT and GIT models, detailed categorisations for all organs can be found in Supplementary Table 2. **E.** Information on model complexity, including the number of studies that included any kind of vascular system, or reported on the oxygenation status of the model. In the pandemic data set, the introduction of immune cells into the model was detected. **F.** The details of the culture conditions reported for relevant models (i.e., models kept in culture) were investigated. Predefined information included manufacturer, supplements, reference, and order number (separate plots for pandemic and post-pandemic data sets can be found in Supplementary Fig. 6). **G.** For stem cell-based models, the detail of the reported differentiation process was extracted, with categories: detailed, reference provided, not detailed, and not reported (Supplementary Table 9, separated plots for pandemic and post-pandemic data sets can be found Supplementary Fig. 7). **H.** For models based on primary material, it was extracted whether any metadata of the donor (biological sex, age, health status) were presented in the studies (separated plots for pandemic and post-pandemic data sets can be found in Supplementary Fig. 8). **I.** Number of investigated areas in the studies indicating how many studies (in %) focused on one, two, or three areas (host factors, infection, and immune response measurements) (separated plots for pandemic and post-pandemic data sets can be found in Supplementary Fig. 9).

The presented data is based on 558 studies of the pandemic (12/2019–02/2022) and post-pandemic period (03/2022–07/2025), categorised according to the investigated organ system and model types (Fig. 2A). Several studies investigated more than one organ system and were included in the analyses per organ. As RT (245 studies) and GIT (83 studies) were the most frequently investigated organ compartments, their outcomes are presented in detail. We categorised the different models into organoids, tissues, cell models, and others (Table 1). Of note is the limited inclusion of studies that exclusively worked on autopsy material.

**Table 1:** Basic definitions of model categories.

Cell models	• 2D culture systems based on primary cells or differentiated stem cells • 3D air-liquid interface systems based on primary cells or differentiated stem cells
Organoids	• Derived from different types of stem cells including adult-, inducible pluripotent and embryonic stem cells • Self-organised 3D tissue-like structures mimicking the modelled organ
Tissue	• Biopsies resected from patients (dead/alive) • Propagated directly or brought into culture (*ex vivo* explants)
Others	• Organ on chip: microfluidic device including living cells mimicking the physiological and mechanical functions of a human organ • 3D bioprinted model: *in vitro* constructs using 3D bioprinting technology depositing layer of bioink including living cells to replicate structures and functions of human tissue

Tissue samples (255 models) and cell models (199 models) were the most frequently used model systems. Organoids (194) constituted a slightly smaller fraction for RT models (28.2%), while for GIT and CNS models, they were the most applied systems with 48.9% and 37.1% respectively (Fig. 2B). The source material and culture types of all models are depicted in Fig. 2C.

#### Respiratory tract

For RT models, we found a similar number of cell culture models, either cultured in 2D (43 models), or as air-liquid interface (ALI) systems (57 models) (Supplementary Table 8). Many tissue models were derived from *ex vivo* biopsy material (64), with a smaller proportion from patients with COVID-19 (autopsy, 24). The RT organoid models were created from either induced pluripotent stem cells (PSC, 31) or adult stem cells (ASC, 39). The corresponding organ compartment, categorised via the description provided by the authors of the manuscript, is displayed for RT models (upper sunburst) and GIT models (lower sunburst) for the pandemic and post-pandemic period (Fig. 2D). While most tissue models (yellow) and organoids (green) mimicked parts of the distal RT (dRT), cell models (blue) represented the proximal RT (pRT) and dRT in almost even parts. It is noteworthy that, for organoids generated from PSCs, the cell type or respective organ compartment is often not specified (categorised then as ‘not specified’). Additionally, tissue samples (yellow) were taken predominantly from the dRT.

#### Gastrointestinal tract

For GIT models, ASC-generated organoids (27) were more common than PSC-based organoids (18) (Fig. 2C, Supplementary Table 8). As already described for RT, the PSC-based organoids were largely not specified for the region or cell type they were supposed to represent (PSC-derived 39% unspecified, ASC-derived 35% unspecified) (Fig. 2D bottom sunburst). Overall, 18.7% of GIT organoids were described to represent the colon. Most tissue models were based on material from patients without COVID-19 (68.7%), predominantly representing the ileum and colon (Fig. 2C and D).

#### Other organ groups

PSCs are used predominantly to form CNS cell models (70.8%), while organoid models (28.3% of all CNS models), are almost exclusively based on PSCs (82.3%) (Fig. 2B and C). A detailed sub-analysis of the different organ compartments was performed for all organs in the pandemic data set (Supplementary Table 8). Despite the prevalence of PSC-based CNS models, there is considerable diversity in the represented brain compartments, ranging from general brain descriptions to specific regions such as cerebral cortex, cerebellum, neuronal, and neural cells (Supplementary Table 8). We categorised the described CNS regions and labelled those we could not specify as “not specified” (36.4% of all PSC-derived CNS models). Four tissue models were generated from *ex vivo* biopsy material from patients without COVID-19, mainly from the cerebral cortex.

Models representing the heart (48) are often tissue or cell models. Only six organoid models were identified in studies investigating the heart. Both organoid models and cell models were generated almost exclusively from PSCs (Fig. 2C).

Similarly, eye models are mostly tissue-based (59.4%) or defined as cell models (24.3%) or organoids (13.5%). Eye cell cultures are often tissue-based corneal and conjunctiva models. Most urogenital tract (UGT) models (68.5%) are tissue-based and represent the placenta. Only 15.0% of the UGT studies in the pandemic data set focused on testicular models (Fig. 2C, Supplementary Table 8). Likewise, kidney models are mostly tissue-based (46.1%), consisting primarily of material from patients without COVID-19 (70%). All kidney organoids are generated from PSCs. Additionally, one organ-on-chip (OoC) kidney model was found. Further specification of kidney compartments was not possible due to a lack of detailed information. The 17 skin models are mostly derived from tissue samples (58.8%), with some representing adipose tissue. Liver studies often apply tissue samples (43.4%) alongside six cell models mainly of hepatocytes, six organoids and one OoC model. All 11 vasculature models represent capillary cells, of which six are PSC-based organoids (Fig. 2C, Supplementary Table 8).

#### Model complexity

The starting material of the different organ models indicates their degree of differentiation and proximity to the original organ. Most models used in these studies represent organ-specific cellular systems, such as epithelial, neuronal, or muscle-based models. Some systems contain several differentiated cell types within the respective lineage but generally lack additional cellular compartments such as immune, stromal, or vascular cells. This reduced cellular complexity facilitates experimental comparability between models. However, few studies employed models of higher complexity that incorporated additional components, such as immune cells or vascular elements. Other studies considered the oxygen content in their model systems (Fig. 2E), which was considered in 11.6% of all GIT models, 8.5% of CNS models, 16% of UGT, 13.4% of kidney models. 1.3% of all RT models, 5% of all UGT models and less than 4% of GIT and eye models included immune cells in their primary cell or organoid models. 6.5% of liver models were identified that used cells to form a vascular structure in their *in vitro* models. Details concerning model complexity were not disclosed in skin studies (Fig. 2E).

#### Quality aspects

Accurate reporting of culture conditions is essential to ensure the reliability of any model. Factors such as the media, manufacturers, batch effects, or supplement composition significantly impact model stability and result replicability as stated in the preprint of the RIVER recommendations.^20^ Here, the level of detail provided on model culture conditions was assessed revealing more than 40% of studies lacking information and only 2.7% of studies reported all four pre-set criteria in the data set of the pandemic period (Supplementary Fig. 6A). Interestingly, data from the post-pandemic period showed an improvement with only 9.3% of studies lacking all information on culture conditions (Supplementary Fig. 6B), which results in an average for both data sets of 31% (Fig. 2F).

As stem cell differentiation is crucial for model formation, we evaluated how the differentiation process for organoids or 2D stem cell-based systems was described in the studies. Detailed information was provided in only 9.9% of studies during the pandemic (Supplementary Fig. 7A). In 23.4% of studies the information provided was rated by reviewers as ‘not detailed’. For the culture conditions, detailed information was found in up to 21.5% of studies in the post-pandemic data set (Supplementary Fig. 7B). The average of both data sets (15.6%) is shown in Fig. 2G and the criteria for these ‘levels of detail’ can be found in Supplementary Table 7.

As variables such as donor biological sex, age and health status are known to influence experimental outcomes, we assessed whether studies reported metadata on patient material which did not differ between pandemic and post-pandemic data sets (Fig. 2H and Supplementary Fig. 8). Many of the studies lacked metadata reporting, with an average of 54.7% across all organ groups failing to provide metadata on the patient source. Among the three key topics—SARS-CoV-2 host factor expression, viral replication, and immune system activation—around 45.1% of the included studies of the pandemic period focused on a single topic, predominantly host factor expression (Supplementary Fig. 9A). Interestingly, analysis of the post-pandemic period revealed an increase in studies investigating all three topics (Supplementary Fig. 9B) from less than 10% in the pandemic data set to more than 25% in the post-pandemic data set, resulting in an average of 20.4% of studies that investigate all three focus areas in their work (Fig. 2I). The results of our meta-research are structured and presented according to research questions 2 to 4, while research question 1 is addressed throughout all sections.

**Fig. 3:**
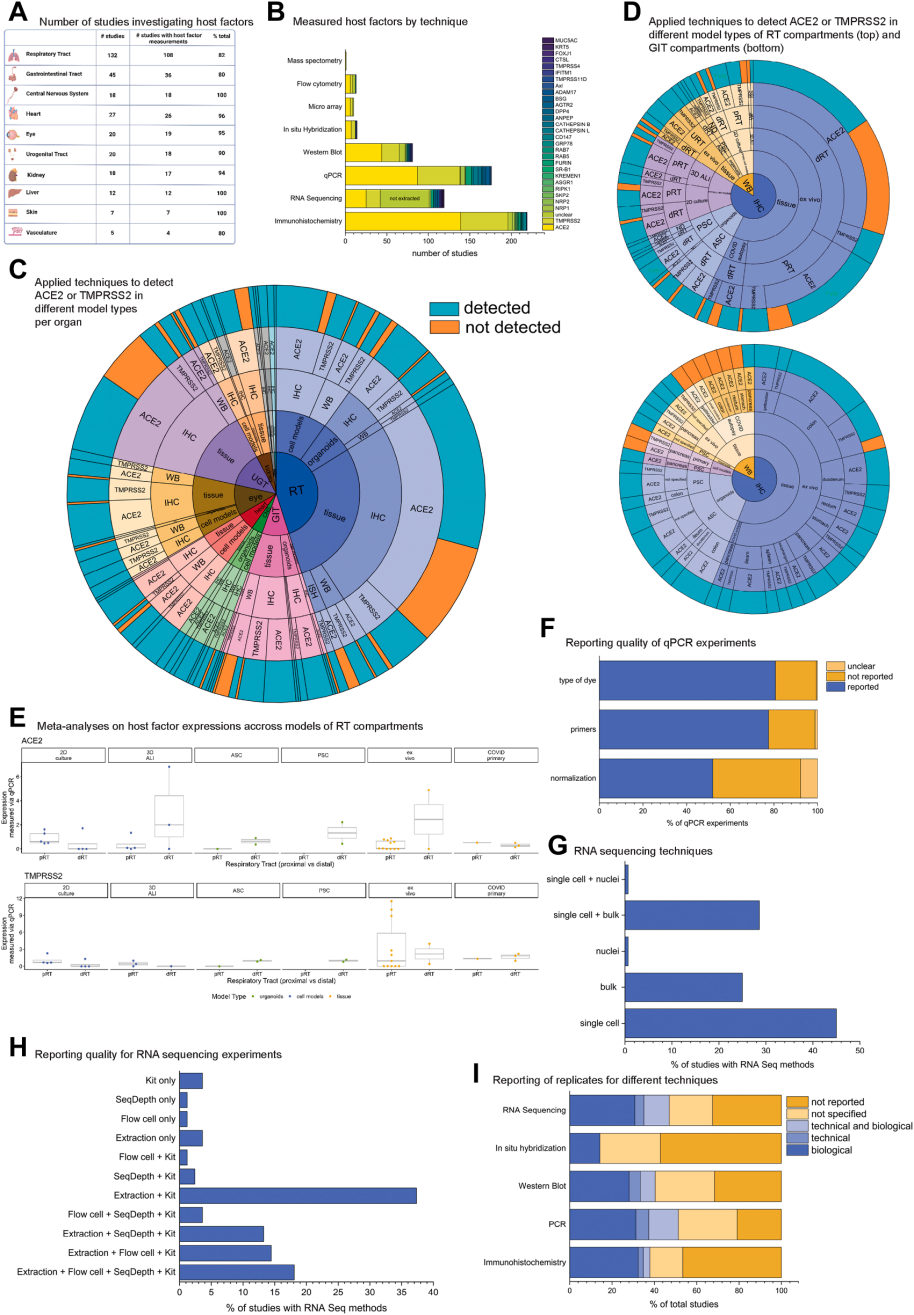
**Summary of host factor measurements in human primary cell, organoid and tissue models. A.** Overview of the number of studies investigating SARS-CoV-2 host factor expression in human organ models. **B.** Summary of all investigated host factors and their detection assays. The dark yellow section in the RNA sequencing bar represents measured host factors for which the reviewers did not extract individual names. **C.** Studies investigating ACE2 and TMPRSS2 across different organ compartments, models, and assays. The reported abundance of these host factors is presented in the outer ring (orange and turquoise). **D.** Distribution of positive and negative detections for ACE2 and TMPRSS2 for the specific source material and described location of RT (1) and GIT (2) models. **E.** Meta-analyses: Top: Expression levels for ACE2 in different organ models for the pRT and dRT based on qPCR results. Bottom: Expression levels for TMPRSS2 in different organ models for the pRT and dRT based on qPCR results. **F.** Reporting quality for qPCR assays averaged of pandemic and post-pandemic data: % of studies reporting dye type, primer sequence, and normalisation method. **G.** Summary of the types of RNA sequencing methods applied in the studies. **H.** Overview of the reported technical aspects of Seq experiments. **I.** Types of replicates reported for each assay (% of total studies), including both host factor and virus detection assays.
• **SARS-CoV-2 infection and/or viral replication** and the associated outcomes on reporting quality are summarised in Fig. 4.

Specifically:

• **Host factor investigations** and the corresponding aspects of reporting quality are detailed in Fig. 3.

**Fig. 4:**
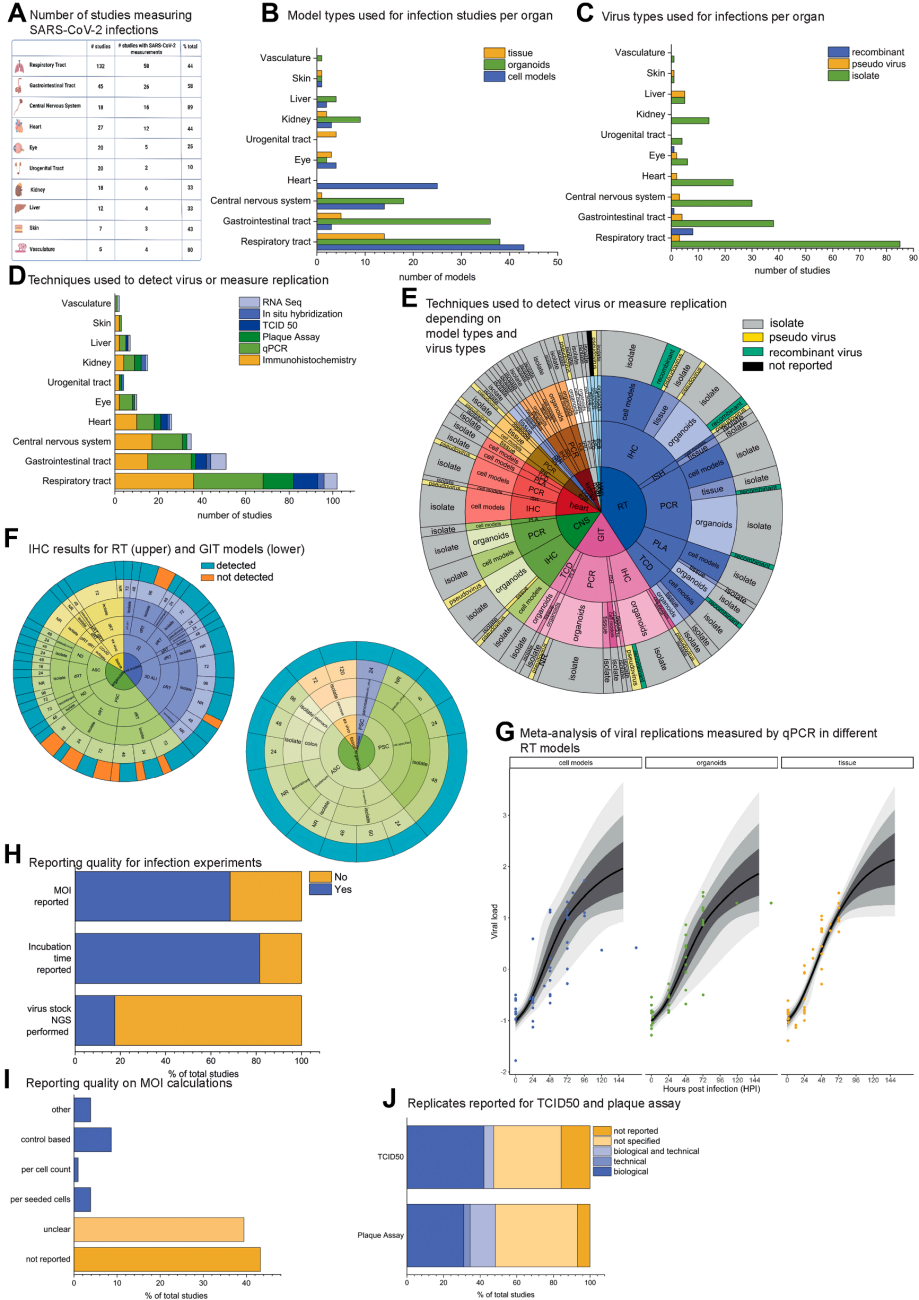
**Investigation of SARS-CoV-2 infections of human organ models displaying the used model types across organ groups, used virus types and summarising the reported outcomes per assay including the respective reporting quality of experimental procedures. A.** Overview of the number of studies investigating SARS-CoV-2, focusing on its detection and/or replication in human models. **B.** Breakdown of the types of models infected, categorised by the organ they represent. **C.** Summary of the different types of viruses used to infect models of various organ compartments. Detailed virus strain information is available in Supplementary Table 13. **D.** Overview of all applied assays used to detect the virus and/or its replication. **E.** Summary of the entire data set, showing which assays were used to detect which viruses in which models. **F.** Analysis of the IHC results for the two dominant organ compartments, RT (1) and GIT (2). The distribution of positive signals (detected, turquoise) to negative signals (not detected, orange) for virus staining is shown in correlation to the virus type, the hours post infection (hpi), the organ compartment, the source material and the model type. **G.** Individual data points and fitted growth curves for viral replication measured by qPCR in RT models. Fitted curves are based on a Bayesian non-linear hierarchical model using a Gompertz function to model viral load increase. Grey areas give 50%, 80%, 95% CI. Viral load was z-transformed with each study and is given in arbitrary units (see methods for details). **H.** Reporting quality questions addressed whether authors performed next generation sequencing (NGS) on the virus stock, reported the incubation time of the virus, and if the virus concentration was reported as multiplicity of infection (MOI) for the pandemic and post-pandemic period. **I.** For the reporting quality the heterogeneity in MOI calculations was assessed. **J.** Also, the types of replicates performed in plaque assay and TCID50 experiments as key infection methods were analysed.
• **Measurements of immune factors** induced by SARS-CoV-2 infections, along with their respective quality reporting criteria, are illustrated in Fig. 5.

**Fig. 5:**
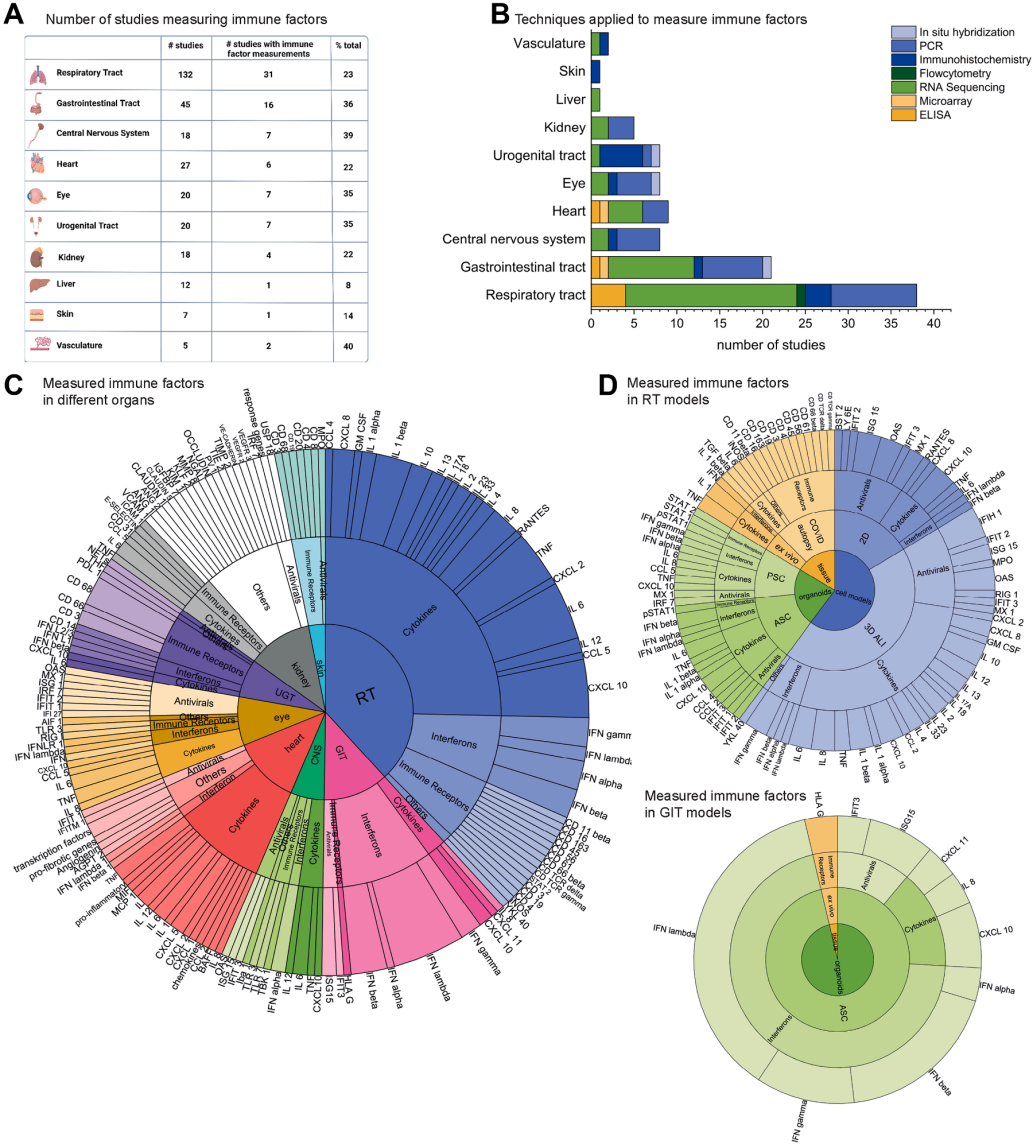
**Immune response in human models after SARS-CoV-2 infection were investigated and summarised according to the represented organ group, applied assays and measured immune factors. A.** An overview of the number of studies investigating the immune response after SARS-CoV-2 infection in the different organ groups. **B.** Overview of all applied assays used to detect immune factors. **C.** Overview of all immune factors that were investigated in the different model types amongst all organs. **D.** For the two dominant organs RT (1) and GIT (2) with the highest number of studies we summarised the immune factors that were measured in the different model types.

### Host factors of SARS-CoV-2

The interaction of viruses with host factors significantly determines pathogenesis. In COVID-19, two host factors for SARS-CoV-2 infectivity, ACE2 and TMPRSS2, were identified early on. ACE2 and TMPRSS2 were established as the primary entry points for SARS-CoV-2, with the viral spike protein binding and priming on the surface of host cells, thereby facilitating viral entry.^21^ However, interactome studies have described numerous other factors that can influence the viral life cycle and disease progression. Understanding the dynamics of host factor expression in different organs provides valuable insights into the mechanisms of infection.^22^ For this reason, many studies have investigated the expression of these early-identified host factors using human 3D models of various organ types (Fig. 2I). Irrespective of the organ compartment, at least 80% of the studies investigated host factors related to SARS-CoV-2 in the pandemic period (Fig. 3A). The most frequently measured host factor was ACE2 in all datasets (blue), followed by TMPRSS2 (yellow) (Fig. 3B). The most applied technique was immunohistochemistry (IHC), with more than 200 studies, followed by qPCR (176), and RNA sequencing (120) (Fig. 3B). Western blot was utilised in 80 studies, less than 20 studies applied in situ hybridisation, micro arrays, or flow cytometry, and only one study used mass spectrometry to measure the expression of ACE2. In addition, many other host factors were measured, although they only account for 1% of the total number of studies. The assays used for the detection of ACE2 and TMPRSS2 were listed, presenting a strong focus on RT, GIT, but also UGT models in the literature (Fig. 3C). ACE2 and TMPRSS2 were predominantly reported positive in the models (ACE2: 77.5%, TMPRSS2: 88.8%). The outcome of the two dominant organs, RT and GIT, and the two most frequently employed protein assays, IHC and WB, were analysed in further detail (Fig. 3D).

#### Respiratory tract

IHC was predominantly used to detect ACE2 in RT models across all three model types. For IHC, 117 measurements of ACE2 were summarised for tissue samples, 26 ACE2 measurements for cell models, and 18 for organoids. ACE2 staining was mostly positive across all RT model types (69.6%), with only a few tissue samples presenting negative staining (orange). TMPRSS2 was measured less frequently, with detection being almost always positive (91.1%). Further examination of host factor analysis by IHC and WB revealed a large proportion of dRT tissue samples, with a focus on ACE2, which was successfully detected in 59.3% of these (Fig. 3D). In the pRT, ACE2 was found positive in 84.4% of studies. TMPRSS2 was almost exclusively found positive in dRT tissue, and in 85.7% of pRT tissue (Fig. 3D).

The RT organoid samples were derived in equal proportions from PSCs and ASCs. For ASC-derived organoids, 8.3% and 14.3% of studies reported negative IHC staining for ACE2 and TMPRSS2, respectively, while in PSC-derived organoids, no negative staining was reported. RT cell models used in IHC experiments were divided into primary 2D cultures (23) and 3D ALI models (15). The 2D models were used to represent the pRT and dRT in almost equal proportions, detecting ACE2 and TMPRSS2 via IHC and WB mostly positively (Fig. 3D). The 3D ALI models primarily represented the pRT (84.6%), with mostly positive IHC and WB staining for ACE2 and TMPRSS2. TMPRSS2 was almost exclusively detected positive in all 2D cell cultures, tissue and organoid models.

#### Gastrointestinal tract

The GIT models were sub-categorised into colon, ileum, jejunum, duodenum, pancreas, stomach, gallbladder, and rectum compartments, or unspecified areas where the studies did not provide sufficient information to determine the exact location represented by the model. IHC was predominantly performed on tissue (37) and organoid models (14) with exclusively positive staining for ACE2 (Fig. 3C). Studies reported missing bands for ACE2 in WB assays in six COVID-19 autopsy samples (Fig. 3D). A 2D cell culture of PSC-derived pancreatic cells showed positive ACE2 signals, whereas a model based on primary pancreatic cells showed negative results for ACE2 and TMPRSS2.

#### Other organ groups

In other organ compartments, IHC was also the dominant method for detecting host factors, with ACE2 being the most frequently measured and often positively detected host factor (77.5%, Fig. 3C). Also, TMPRSS2 was reported almost exclusively positive, irrespective of organ or model type (88.8%). We identified absent IHC staining for ACE2 in 30% of measurements in UGT tissue models. In the whole data set of 24 CNS models, we found only one absent ACE2 staining.

#### Meta-analysis

For the meta-analysis, we extracted data points from qPCR plots detecting the expression of SARS-CoV-2 host factors ACE2 and TMPRSS2 in RT models. Our objective was to conduct a meta-analysis of ACE2 and TMPRSS2 expression across different model systems by using a Bayesian hierarchical model to document the expression patterns in the different model types and display potential correlations between different organs. Due to the low number of data points, meta-analyses could only be conducted for RT data.

qPCR data were reported in a heterogeneous manner with different housekeeping genes and inconsistent reporting on normalisation approaches (Supplementary Table 4). To account for these differences between studies, we modelled study and housekeeping gene as a random effect in our Bayesian hierarchical model to account for baseline differences between studies. Posterior distributions of model parameters were used to estimate parameters, credible intervals and to test hypotheses regarding differences in host factor expression between experimental models, for pRT and dRT, and their respective interactions. Estimated effects and credible intervals for experimental model and RT location were not indicative of meaningful differences between any groups or interactions (all credible intervals contain 0 with the exception of the intercept in the ACE2 model; Supplementary Tables 10 and 11, Supplementary Fig. 2) with considerable part of the variance explained by the study (ACE2: I^2^ = 61.7%; TMPRSS2: I^2^ = 74.8%; Supplementary Tables 14 and 15). There were no differences between organ models regarding their expression of either ACE2 or TMPRSS2. Moreover, there were no detectable differences between expression in the upper and lower respiratory tract (descriptive data Fig. 3E).

#### Quality aspects

To assess whether reporting quality improved from the pandemic to the post-pandemic period, we analysed relevant data and found that qPCR studies reported dye type and primer details in 77.5% of cases on average, with no difference between the two periods (Fig. 3F). However, 40.2% of studies did not report the normalisation method used for qPCR data and 7.8% reported it unclearly. For the meta-analysis, some studies had to be excluded since expression levels could not be extracted due to normalisation of controls to 100%.

Since RNA sequencing data sets require specialised domain knowledge, their extraction was handled separately by a team of RNA sequencing experts. The different types of RNA sequencing were summarised: Of all applied RNA sequencing measurements, 45.0% exclusively applied single-cell RNA (scRNA) sequencing, 28.6% combined scRNA with bulk sequencing, 0.7% employed single-nuclei RNA (snRNA) sequencing, and 25% used only bulk sequencing (Fig. 3G). To assess the quality of reporting for RNA sequencing methodologies, we examined how well essential technical aspects were documented in the manuscripts with 18.1% of studies reporting all predefined details (Fig. 3H, further quality control aspects are shown in Supplementary Fig. 3).

Reporting the sample size *in vitro* experiments is crucial for readers to assess both the reliability of results and the level of inference. A larger sample size, particularly when using biological replicates, enhances the statistical power leading to more reliable results. For the assessment it is equally important to distinguish between the types of replicates used. Technical replicates, derived from the same source material, help assess the precision of the experimental procedure itself by controlling for technical variability. However, biological replicates, originating from different biological sources, are essential for capturing the natural variation within a population and drawing broader inferences.

The reported sample sizes across all evaluated assays and measurements are summarised in Fig. 3I. For WB, 28.1% of studies reported biological replicates, 5.3% reported technical replicates, and only 7.0% reported both, while 31.6% did not report any replicates. In IHC assays, 32.3% of studies with biological replicates were detected, and 46.6% of studies did not report any replicates. 13.9% of studies reported biological and technical replicates for qPCR experiments, and 31.3% reported only biological, 6.1% only technical replicates. In another 27.8% of qPCR data sets, the type of replicates was not specified, and 20.9% did not report replicates at all. In 32.5% of the studies using RNA sequencing, it was not reported if the experiment was replicated, or multiple samples were used. In 20.5% of studies, replicates were mentioned but not specified. 30.8% used biological replicates, often in larger studies focusing solely on sequencing patient material.

### SARS-CoV-2 infections

The detection of replicated virions after a SARS-CoV-2 infection in the different organ systems is one of the most important assays for understanding COVID-19 pathogenesis. However, infection and detection require access to BSL-3 laboratories, which limits their execution. In the pandemic period SARS-CoV-2 infections were performed in 44% of RT studies (Fig. 4A). In GIT studies, 58% included SARS-CoV-2 infections. Nearly all CNS studies (89%) and VASC system studies (80%) induced infections and/or measured SARS-CoV-2 in the models. In all other organ groups, less than 50% of the studies described infections.

#### Respiratory tract

In RT research, 2D cell cultures were the most applied, accounting for 43 infected models, followed by organoids (38) and tissue models (15) (Fig. 4B). Most studies utilised SARS-CoV-2 isolates (85) with USA/WA1/2020 being the most prevalent strain (Fig. 4C, Supplementary Table 2). Some studies used recombinant viruses (8), and a small proportion of studies reported the usage of pseudo viruses (3). Immunohistochemistry (36) was predominantly used in studies investigating infections of the RT, compared to studies in other organ compartments.

The IHC results of RT models were summarised in more detail, including model types and sources, represented sub-compartments, virus types, sampling time points, and reported outcomes (detected or not detected) (Fig. 4F). Notably, RT models predominantly reported positive signals, especially in ASC-derived organoids and tissue, regardless of location or time point.

#### Gastrointestinal tract

As described above, studies around the GIT are the only ones that predominantly used organoids to study SARS-CoV-2 infections (81.8%) (Fig. 4B). Most studies applied virus isolates (38) for their infections (Fig. 4C). Among the GIT studies, qPCR (20) and IHC (15) were the most used assays to detect the virus or measure its replication (Fig. 4D and E). The extraction of the experimental results obtained by IHC for virus detection shows that irrespective of the model, applied virus type and time point of measurement after infection, the staining for the virus was positive (Fig. 4F).

#### Other organ groups

All three model types were employed across organ compartments to study infection, except in heart and UGT studies, where no organoids were applied (Fig. 4B). Overall, primary cell or stem cell-based cultures were the dominant model type used for infection studies, with only CNS, kidney and liver studies predominantly using organoid models (Fig. 4D). Isolates were the primary virus type used for infection across studies. IHC was the most frequently used assay, providing visual confirmation of infection (total 90) (Fig. 4D and E). *In situ hybridisation (9)* and RNA sequencing (20) were performed in only a few studies. Assays measuring viral replication, such as plaque assays (26), tissue culture infection dose (TCID50; 20), and qPCR, constituted half of all assays used, with qPCR (total 90) being almost as commonly used as IHC in all organ groups.

#### Meta-analysis

We compared viral replication in RT models, cell models, organoids and tissue of dRT and pRT within 0–144 h post infection. The meta-analysis was based on a Gompertz growth model fit by a Bayesian non-linear hierarchical model. We specifically investigated the viral replication reported for the three different model systems in the RT (Fig. 4G). Here, we extracted the data from reported replication curves detected by viral PCR. The aim was to compare the viral load over time between the different models. Viral PCR data were heterogeneously reported (Supplementary Table 5). Estimated effects and their credible intervals were not indicative of meaningful differences in viral replication speed between model types within the analysed data set (all pairwise comparisons between *ex vivo*, organoids, and tissue had a posterior probability >0.29, Supplementary Table 13). As the included studies used different virus isolates and variants, these results should be interpreted as a comparison of reported replication patterns across model systems rather than a direct comparison of intrinsic viral growth kinetics. This is also reflected in the very large proportion of variance explained by individual studies (I^2^ = 99%; Supplementary Table 16). Notably, all model types showed similar positive increases in viral load after infection with a maximum replication rate at approximately 60 h post infection (Fig. 4G, Supplementary Table 13).

#### Quality aspects

The plotting of qPCR results for viral replication was assessed as the key assay to analyse infection assays. It showed that 24-, 48-, and 72-h post-infection were the most measured time points. The plotting of the data was reported on a broad set of different y-axes (Supplementary Table 5). The incubation time of the virus on the samples was reported in 81.5% of all infection studies in the pandemic and post-pandemic data sets combined (Fig. 4H). Only a small percentage (17.6%) of studies performed next-generation sequencing on the virus strain as a quality measure to avoid unaccounted genetic mutations. 68.5% of studies reported the applied viral concentration in terms of multiplicity of infection (MOI). Other studies used different units or did not report the concentration at all. Among those studies that reported MOI, 39.4% did not provide clear explanations on MOI calculations, and another 43.3% did not report their calculations at all. 13.5% of studies provided details on MOI calculations based on controls, seeded cells, or cell count (Fig. 4I). During infection studies, mainly biological replicates were used in TCID50 and plaque assays. More than 50% of studies using TCID50 and plaque assays did not mention the sample size or specify whether biological or technical replicates were used (Fig. 4J). For the plaque assay, we extracted whether the studies provided an image of the formed plaques in the petri dishes, which none of the studies of the GIT, eye, and UGT organ groups did and only 5% of the RT studies, 50% of the CNS, and 33% of the heart and kidney studies did. For the viral qPCR data, almost all studies reported the used primers (Supplementary Fig. 5).

### Immune responses after SARS-CoV-2 infection

In addition to analysing host factor expression and the ability of SARS-CoV-2 to replicate in various organ systems, the mostly anti-viral immune response induced by the virus is another key aspect that has been addressed in numerous studies. Immune responses to SARS-CoV-2 infections were analysed in 27.0% of studies (Fig. 5A), primarily via RNA sequencing or qPCR (Fig. 5B). As it was not methodologically possible to extract the actual quantitative results of the measurements, only the measured immune factors were summarised. Here, we present all immune factors, across organ groups, summarised in categories (cytokines, interferons, immune receptors, antivirals and others).

#### Respiratory tract

For studies investigating the RT, 23.5% performed experiments on the immune response after infection of the respective model (Fig. 5A). The most used test to detect immune factors was RNA sequencing (43), followed by qPCR (33), with only a low number of studies reporting the immune factors on protein level (Fig. 5B). Overall, cytokines were the most measured immune factors in RT models (66.3%) (Fig. 5C). These studies primarily employed 3D ALI models (45.5%), followed by organoids (23.4%) and 2D primary cell cultures and tissue models (16.4% each) (Fig. 5D). Tissue samples from the RT were mainly sourced from patients with COVID-19 (76.2%) and often investigated for immune receptors.

#### Gastrointestinal tract

In GIT studies, 36% (16 studies) analysed immune factors, primarily applying RNA sequencing (10) and qPCR techniques (7) (Fig. 5A and B). Notably, of all measurements 70.4% focused on interferons in GIT models, with less focus on cytokines as compared to RT models (Fig. 5C). A detailed overview of the model types used to investigate immune factor expression after SARS-CoV-2 infection in the GIT shows that ASC-derived organoids served almost exclusively to study immune factors in GIT models, besides one tissue model investigating an immune receptor (Fig. 5D).

#### Other organ groups

Immune factors were investigated in 38.9% of all CNS studies, primarily using qPCR (5), and with a strong focus on cytokines (35.7%). Also, for eye, UGT and VASC models more than 35–40% of studies investigated several immune factors, while studies around kidney, liver, skin and heart models were less focused on immune factors (<25%) (Fig. 5A). For UGT and skin models, most of the reported immune factors fell into the immune receptors category. For all other organ groups, cytokines were the most frequently measured immune factors. Only for kidney models, studies presented several immune factors that fell into the category ‘Others’, where reviewers could not categorise the factors into the predefined categories (Fig. 5C).

## Discussion

Advanced human culture model systems have become indispensable tools in biomedical research and already provide insights into the physiological and pathological aspects of various diseases.^1^^,^^23^ They are particularly relevant in infectious diseases such as COVID-19, where they enable stratification of viral pathogens and variants based on replication capacity, cellular tropism, and early immune responses. The COVID-19 pandemic created a setting for the rapid expansion and application of such models, allowing a systematic evaluation of their contribution to addressing shared research questions within a short time frame. To capture temporal developments, we analysed two consecutive data sets: an early pandemic phase (December 2019–February 2022; n = 292) and a subsequent phase (March 2022–July 2025; n = 266). This division reflects a transition from an initial surge of COVID-19 research to a more consolidated phase beginning in early 2022, as suggested by bibliometric, thematic, and epidemiological trends, despite the continued public health emergency.^24^, ^25^, ^26^, ^27^

Based on early clinical evidence of extra-pulmonary involvement in SARS-CoV-2 infection,^28^ a wide range of research groups applied available model systems to investigate host factors, viral replication, and immune responses. Consequently, the diversity of models analysed in this SR likely reflects availability rather than suitability. A strong focus on respiratory tract (RT) models (41.8%) was observed, likely driven by the classification of SARS-CoV-2 as a respiratory virus and the availability of suitable infection systems, while GIT and CNS models accounted for 14.1% and 9.4%, respectively, with stable distributions across both study periods. Across model types, host factor analysis–primarily ACE2 and TMPRSS2–dominated, while viral replication and immune responses were assessed less consistently, which may partly be related to limited access to BSL-3 facilities.

Model systems frequently represented distinct anatomical compartments, with cell models predominantly reflecting the proximal respiratory tract (pRT) and organoids the distal respiratory tract (dRT), while GIT organoids modelled defined segments such as colon or ileum. Although this compartmentalisation may improve physiological specificity, it can reduce comparability across models within the same organ. Meta-analyses did not reveal differential host factor expression or viral replication between models. Results have, however, low generalisability as between study heterogeneity was substantial in all models. In the post-pandemic data set, studies more frequently combined viral replication, host factor analysis, and immune responses, indicating increased analytical depth while the overall distribution of model types remained stable.

Despite these advances, important conceptual and biological limitations remain. In many models, particularly PSC-derived systems, the exact organ compartment or cell type was insufficiently specified, limiting interpretability and reproducibility.^29^ Inadequate model annotation and inconsistent nomenclature hinder comparability across studies, emphasising the need for standardised frameworks to define cell identity and differentiation status (Key Recommendation 1 in Panel 1: Seven Key Recommendations). Detailed reporting of model characteristics is essential, particularly given the variability inherent to stem cell-based systems.^30^ Moreover, most models focus on single cell types, such as epithelial, muscle, or neuronal cells, while more complex systems incorporating immune or vascular components remain rare. This reflects a fundamental trade-off between experimental simplicity and physiological relevance, as increased complexity may enhance biological insight but complicates standardisation (Key Recommendation 4 in Panel 1).

Panel 1 **Seven key recommendations.**

1. **Develop a standardised nomenclature and quality control framework** for identifying stem cell source material, differentiation status, and cell/tissue identity to enhance clarity and comparability across studies.
2. **Ensure transparency and reproducibility** through clear reporting of experimental units, replicates, culture conditions, and infection parameters, supported by sufficient resources for detailed methods documentation.
3. **Demonstrate proof of model suitability** by validating infection models with relevant host factors, viral replication data, and control standards to ensure robust and meaningful results.
4. **Enhance physiological relevance of human organ models** to better replicate *in vivo* conditions, improving their predictive value for human health and disease.
5. **Improve and enforce reporting standards in scientific publications**, ensuring comprehensive documentation and consistency across human organ model research.
6. **Encourage transparent data practices** through accessible raw data sharing and publication of both negative and inconclusive results to reduce reporting bias and broaden scientific insight.
7. **Foster interdisciplinary research networks** to pool domain expertise, from organ model generation to infection studies and data analysis.

Improving the physiological relevance of human organ models requires not only increasing structural complexity but also systematically evaluating how well these systems recapitulate disease processes observed in patients (Key Recommendation 4 in Panel 1). Several studies have begun to address this by linking organoid findings to clinical observations and patient-derived data sets. For example, SARS-CoV-2 organoids reproduce infection patterns and host factor expression profiles observed in patient tissues and transcriptomic data.^5^^,^^31^ More direct cross-validation has been demonstrated by matching transcriptional responses in infected kidney organoids with proteomic signatures from urine of critically ill patients with COVID-19.^32^ Beyond infectious diseases, patient-derived organoid biobanks in oncology illustrate how functional responses in organoid drug screens have been reported to correlate with patient treatment outcomes.^33^ Together, these approaches highlight that validation against host factor expression, viral replication, and clinically relevant readouts is essential to demonstrate model suitability and predictive value (Key Recommendation 3 in Panel 1). While human organ models hold substantial promise for antiviral drug discovery and therapeutic testing,^5^ our findings indicate that current limitations in standardisation, reproducibility, and reporting remain major barriers. Addressing these challenges through harmonised protocols, quality control, and systematic benchmarking will be essential to enable a robust bidirectional translational framework linking experimental models and clinical observations (Panel 1: Key Recommendations 1, 5, 6).

A central finding of this SR is that limitations in reporting and standardisation represent a major barrier to evidence synthesis and translation. Reporting quality was frequently insufficient, although improvements were observed in the post-pandemic dataset, with missing information on culture conditions decreasing from >40% to <10%. However, key issues remained, including unclear definitions of experimental units, insufficient distinction between biological and technical replicates, and incomplete reporting of donor metadata such as age, sex, and health status.^34^ In addition, negative results were rarely reported, suggesting potential reporting bias.^35^^,^^36^ Systematic assessment of such biases through for example funnel plots was, however, not possible as information on study variance was often missing. These shortcomings directly limit interpretability and generalisability across studies and highlight the need for transparent and comprehensive reporting practices (Key Recommendations 2, 5, 6 in Panel 1).

These reporting deficits substantially affected data integration and meta-analysis. Data extraction required substantial manual effort and often relied on estimations from plotted data due to the lack of accessible raw values. Missing key parameters, including delta Ct values, virus concentration, incubation time, controls, and replicates, combined with low adherence to PCR reporting standards (MIQE),^37^ limiting comparability across studies. In particular, normalisation strategies frequently rendered results non-comparable, leading to exclusion of data sets and reducing statistical power. Consequently, meta-analytical findings should be interpreted as preliminary and exploratory. These limitations underscore that robust evidence synthesis in human *in vitro/ex vivo* research critically depends on standardised reporting, accessible raw data, and consistent methodological frameworks (Key Recommendations 2, 5, 6 in Panel 1).

More broadly, these findings highlight a fundamental challenge: while reporting shortcomings may be tolerable for individual studies, they critically impair evidence synthesis. Addressing complex research questions, such as differences in viral replication across organ compartments, requires systematic reviews and meta-analyses, which provide higher levels of evidence than individual studies.^38^^,^^39^ For human organ models to fulfil their translational potential and support the reduction of animal experiments, they must be valid, reproducible, and accessible.^29^^,^^40^, ^41^, ^42^, ^43^ Achieving this requires transparent methodological reporting and harmonised standards as a foundation for cumulative evidence generation (Key Recommendations 2, 5 in Panel 1).

This SR also contributes to the emerging field of meta-research *in vitro* systems. While most existing SRs focus on single models and relatively small data sets,^11^, ^12^, ^13^, ^14^, ^15^, ^16^ our analysis integrates 558 studies to assess how diverse human organ models address shared research questions within a common disease context. Ongoing efforts aim to develop automated data extraction tools and standardised guidelines for evaluating *in vitro* studies.^41^ Despite this progress, the lack of standardisation currently limits the integration of individual studies into a coherent evidence base. Strengthening reporting standards and methodological consistency is therefore essential not only for scientific rigour but also for enabling regulatory acceptance and broader application of human organ models.

This study has several limitations related to the novelty and complexity of the approach. Studies based solely on autopsy material were excluded for feasibility reasons. A lack of standardised nomenclature and inconsistent reporting complicated model classification and data extraction. Despite structured protocols, approximately 60% of extracted data required reconciliation. Incomplete reporting of replicates, variance, and key experimental parameters limited data availability and affected the robustness of the meta-analyses, resulting in conservative estimates. These limitations further underscore the need for improved standardisation and reporting practices.^20^^,^^37^^,^^44^, ^45^, ^46^

The COVID-19 pandemic highlighted the need for robust and rapidly deployable research strategies to support evidence-based decision-making. These findings have direct implications for future pandemic preparedness. Rapid deployment of human organ models will require pre-established standards for model characterisation, reporting, and data integration to ensure comparability and actionable results across laboratories (Key Recommendations 1, 5 in Panel 1). In parallel, systematic integration with clinical data will be essential to translate mechanistic insights into risk assessment and therapeutic strategies (Key Recommendations 3, 4 in Panel 1).

Interdisciplinary research networks provide an effective framework to achieve this. For example, the Organo-Strat network within the German Network of University Medicine (NUM)^47^ enabled coordinated analyses across model systems through standardised protocols and collaboration between model developers and infection biology experts. Such approaches facilitate the integration of expertise from model generation to data analysis and strengthen the translational relevance of human organ models (Key Recommendations 7 in Panel 1).

To fully realise this potential, improved reporting standards, transparent data practices, and coordinated research efforts are essential. Together, these measures will enable human organ models to serve as robust, scalable platforms in future pandemic research and the preclinical pipeline.

## Contributors

All authors read and approved the final version of the manuscript. Conceptualisation: MH, SKN, AL, MB, KH, VFV, NID, TS, ABB, LGTA, HS, SHip, UT, ACH; Data Curation: MH, SKN, MA, SKH, ISD, CK, ACH; Formal Analysis: MH, SKN, UT; Funding Acquisition: MH, UT, ACH; Investigation: MH, SKN, NID, UT, ACH; Data Collection: MH, SKN, AL, MB, KH, VFV, NID, TS, ASMA, MA, LB, AF, DF, MF, LH, SaH, MK, SKH, CK, HK, SKU, JK, KAL, PM, SSS, SS, LGTA, EW, HS, SHip, ISD; Methodology: MH, SKN, ABB, BGC, UT, ACH; Project Administration: MH, SKN; Resources: ACH; Software: BGC, UT; Supervision: MH, SKN, UT, ACH; Validation: MH, SKN, ACH; Data Visualisation: MH, SKN, SS, UT; Writing Original Draft: MH, SKN, UT, ACH; Writing- Review & Editing: MH, SKN, AL, MB, KH, VFV, NID, TS, ASMA, MA, ABB, LB, AF, DF, MF, LH, SaH, MK, BGC, SKH, CK, HK, SKU, JK, KAL, PM, SSS, SS, LGTA, EW, HS, SHip, UT, ACH.

SKN, UT and MH have verified the underlying data.

## Data sharing statement

This SR was preregistered on the International Prospective Register of Systematic Reviews (PROSPERO, CRD42021250999). The search strategy, data extraction protocol, libraries of all studies separated by organ groups, coding of the meta-analyses and the GSE numbers of the RNA sequencing data sets are made available on OSF under a CC-BY licence.^15^

## Declaration of interests

NID is an external advisor and animal welfare officer for Medizinisches Kompetenzzentrum c/o HCx Consulting GmbH (Medizin im Grünen) in Brandenburg. PM received travel expenses from UCB.

The other authors declare no competing interests.
