# Supplementary material for: Low reporting quality limits the contribution of human organ models to COVID-19 research: a systematic review
Source: eBioMedicine. 2026 Jul 28;130:106392. doi: 10.1016/j.ebiom.2026.106392 (PMC13449789; doi:10.1016/j.ebiom.2026.106392)
Supplement: Supplementary file 1 [file mmc1.pdf]

Table of Contents

Supplementary Table 1: Used acronyms in this study 2
Supplementary Table 2: Search term strategy 3
Supplementary Table 3: Eligibility criteria 4
Supplementary Table 4: Overview of the different y-axes for host factor qPCR results 5
Supplementary Table 5: Overview of the different y-axes for viral qPCR, Plaque Assay and TCID50 results 6
Supplementary Table 6: Sensitivity Analysis: Bayesian Hierarchical Model estimates for ACE2 expression level in the different model systems and respiratory tract: cell cultures, organoids and tissue. 8
Supplementary Table 7: Sensitivity Analysis: Bayesian Hierarchical Model estimates for TMPRSS2 expression level in the different model systems and respiratory tract: cell cultures, organoids and tissue. 9
Supplementary Table 8: Organ groups with models and locations 9
Supplementary Table 9: Explanation of the answer options to describe the differentiation of stem cells 13
Supplementary Table 10: Bayesian Hierarchical Model estimates for ACE2 expression level in the different model systems and respiratory tract: cell cultures, organoids and tissue........................ 13
Supplementary Table 11: Bayesian Hierarchical Model estimates for TMRSS2 expression level in the different model systems and respiratory tract: cell cultures, organoids and tissue. 14
Supplementary Table 12: Overview of the different virus strains 14
Supplementary Table 13: Results of meta-analysis on viral replication in respiratory tract models 15
Supplementary Table 14: Heterogeneity Measure from Bayesian Hierarchical Model for PCR ACE2 16
Supplementary Table 15: Heterogeneity Measure from Bayesian Hierarchical Model for PCR TMPRSS2... 16
Supplementary Table 16: Heterogeneity Measure from Bayesian Hierarchical Model for viral infection... 16
Supplementary Methods 1: Bayesian Modelling of Host Factor Expression in Respiratory Tissues 17
Supplementary Methods 2: Bayesian Modelling of Infection Curves Using Gompertz Function..... 18
Supplementary Methods 3: Protocol Development and Preregistration 19
Supplementary Methods 4: Systematic Literature Search Strategy and Study Selection 19
Supplementary Methods 5: Training.. 20
Supplementary Methods 6: Data Collection, Data Handling and Analysis 20
Supplementary Methods 7: PRISMA checklist 20
Supplementary Figure 1: Extraction of observations for meta-analyses for host factors ACE2 and TMPRSS2... 24

Supplementary Figure 2: Posterior predictive plots for meta-analyses on host factors for ACE2 (upper panel) and TMPRSS2 (lower panel). 25
Supplementary Figure 3: Metadata and Quality Control in RNA Sequencing experiments 26
Supplementary Figure 4: Plaque Assay Images 26
Supplementary Figure 5: Primer Sequence 27
Supplementary Figure 6: Reported details on culture conditions for models during the pandemic (A) and post-pandemic period (B) 27
Supplementary Figure 7: Reported details on stem cell differentiation during the pandemic (A) and post-pandemic period (B) 28
Supplementary Figure 8: Reported metadata for patient derived material during the pandemic (A) and post-pandemic period (B) 29
Supplementary Figure 9: Number of investigated areas per study during the pandemic (A) and post-pandemic period (B) 29
Supplementary Figure 10: Forest plot based on posterior draw of ACE2 brms model (see also Supplementary Table 10) 30
Supplementary Figure 11: Forest plot based on posterior draw of TMPRSS2 brms model (see also Supplementary Table 11) 31

Supplementary Table 1: Used acronyms in this study

**Acronyms**	
ACE2	Angiotensin-Converting Enzyme 2
ALI	Air-Liquid Interface
ASC	Adult Stem Cells
CNS	Central Nervous System
COVID-19	Coronavirus Disease 2019
dRT	distal Respiratory Tract
GIT	Gastrointestinal Tract
HF	Host Factor
HPI	Hours Post Infection
IHC	Immunohistochemistry
ISH	In Situ Hybridization
MDAR	Material Design Analysis Reporting (checklist)
PCR	Polymerase Chain Reaction
PRISMA	Preferred Reporting Items for Systematic Reviews and Meta-Analyses
PROSPERO	Prospective Register of Systematic Reviews

pRT	proximal Respiratory Tract
PSC	Pluripotent Stem Cells
qPCR	Quantitative Polymerase Chain Reaction
RoB	Risk of Bias
RNA SEQ	RNA Sequencing
RT	Respiratory Tract
SARS-CoV-2	Severe Acute Respiratory Syndrome Coronavirus 2
SRs	Systematic Reviews
TCID50	Tissue Culture Infection Dose50
TMPRSS2	Transmembrane Serine Protease 2
UGT	Urogenital Tract
VASC	Vasculature
WB	Western Blot
WHO	World Health Organization

Supplementary Table 2: Search term strategy

To ensure search term completeness, a systematic concept was developed. We identified synonyms and Boolean operators that were applied for the specific database.

Search Term Concept PubMed Database	
**Concept 1: Disease**	Keywords: COVID-19, SARS-CoV-2
**Concept 2: Model System**	Keywords: human, 3D tissue culture, explants, organoids, organ-on-a-chip, spheroids
**Concept 3: Host factors of SARS-CoV-2**	Keywords: ACE2, Furin, Basigin, CD147, TMPRSS2, NRP1, AXL, protein expression
**Concept 4: Organisms**	Keywords: NOT animals, NOT humans
**Concept 5: Language**	Keywords: English
**Concept 6: Publication type**	Keywords: NOT review, NOT systematic review
Serch Term Concept WHO database	
**Concept 1: Model System**	Keywords: human, 3D tissue culture, organoids, organ-on-a-chip, spheroids, autopsy, explant, pathology

**Concept 2: Host factors of SARS-CoV-2**	Keywords: ACE2, Furin, Basigin, CD147, TMPRSS2, NRP1, neuropilin, AXL, protein expression
**Concept 3: Publication type**	Keywords: prognostic studies, risk factor studies, etiology studies, diagnostic studies, observational studies, guidelines, clinical trials, case reports, incidence studies, policy brief, screening studies, prevalence studies, qualitative research, evaluation studies, experimental studies, RCT, article

Supplementary Table 3: Eligibility criteria

Studies were screened for eligibility according to the listed inclusion and exclusion criteria during the full text screening.

INCLUSION CRITERIA
• Primary research article
• Research article written in English
• Study using human material (cells or tissue)
• Study using dissected tissue from patients (dead or alive) either natively used (e.g., for histology) or brought into culture
• Study directly extracting single cells from patient material for further relevant experiments
• Study performing relevant experiments on human tissue or 3D cell culture models (organoids, explants, etc.)
• Study performing relevant experiments on 2D cultures of primary human cells (primary cells can be extracted from patients, tissue or commercially acquired)
• Study performing relevant experiments on 2D cultures of differentiated human stem cells
• Study analyses SARS-CoV-2 host factor expression on human tissue or eligible human cell culture models
• Study analyses SARS-CoV-2 virus replication or accumulation in human tissue or eligible human cell culture models
• Study analyses immune responses or inflammatory processes related to SARS-CoV-2 infection in human tissue or eligible human cell culture models
EXCLUSION CRITERIA
• Non-primary research article: Publications without primary data (e.g., reviews / perspectives / opinion papers, conference abstracts)
• Study presents exclusively experiments on non-human material (e.g., mouse)
Experiments performed on non-human tissue or non-human cell culture models
• Xenograft data
• Data on cancer cell lines
• Data on standard 2D cell lines (293, Huh7, HUVEC, Caco, HepG2,....)
• Experiments performed on blood samples (incl. PBMCs)
• Study presenting data based on previously published data sets or databases (in silico) (RNA SEQUENCING data are an exception)
• Clinical study (therapy, vaccine or drug treatments without data on non-treated human tissue or eligible human cell culture models)

Supplementary Table 4: Overview of the different y-axes for host factor qPCR results

The y-axes for qPCRs measuring host factor expression were extracted showing a large variety. In 300 figures plotting qPCR data, 52 different y-axes were displayed.

PCR y-Axes
2^-delta Ct
ACE2 (fold change)
ACE2 mRNA expression
ACE2/PPIA
ADAm17 (fold change)
AGTR2 (fold change)
ANPEP (fold change)
BSG (fold change)
CATHEPSIN B (fold change)
CATHEPSIN L (fold change)
delta Ct (GOI-GAPDH)
delta Ct (vs. POLR2A)
DPP4 (fold change)
fold change
fold change above iPCS
fold change in TMPRSS2 mRNA over GAPDH
fold change normalized to untr.
fold change vs. hESCs
fold expression change (2^delta delta Ct)
fold increase in ACE2 mRNA
fold inrease vs only medium
Fold over HEK293T RNA
gene expression /10^6 GAPDH (log10)
gene expression fold change
Log10 (fold change)
mRNA expression (AU)
mRNA expression level (per 10^6 GAPDH, log10)
mRNA expression relative to PPIA
mRNA expression relative to TBP
mRNA level (% of GAPDH)
mRNA level relative to GAPDH
mRNA transcripts relative to GAPDH
normalized expression (log2 dCT)
normalized expression relative to alvelospheres
normalized mRNA expression
Normalized relative expression
Relative ACE2 expression

relative ACE2 mRNA expression
relative basal ACE2 mRNA expression
relative expression
Relative expression normalized to GAPDH
relative Furin mRNA expression normalized to GAPDH mRNA
relative gene expression
relative hACE2 mRNA level (normalized to GAPDH)
relative mRNA expression
Relative mRNA fold change
relative mRNA fold change of TMPRSS2
relative normalized expression
Relative RNA Level
Relative to GAPDH
TMPRSS2 (fold change)
TMPRSS2/PPIA

Supplementary Table 5: Overview of the different y-axes for viral qPCR, Plaque Assay and TCID50 results

The y-axes for plots showing qPCR, Plaque Assay or TCID50 results measuring viral replication or viral load were extracted. Within every assay we detected a large variety in the way data were plotted.

viral PCR y-axes
% in detected viral copies
% SARS-COV-2 GEs
(N) fold change
Arbitary units
copies of SARS-COV-2 (RNA log10)
copies/µl of SARS-COV-2
Ct cov-N- Ct GAPDH
delta CT
fold change
fold change cov2 genome
fold change to 0h
genome equivalents/mL (log 10)
genomic copies /µL (log 10)
Infectivity (relative to peak)
Log 10 TCID50 eq/ml
log 10 viral RNA level
normalized relative expression
Normalized relative expression
Normalized viral sgRNA
ORF-1b gene copies
qPCR (relative value)

RdRp gene copies, 1x10^6
relative fold change SARS-COV-2
Relative fold change SARS-COV-2
relative fold change SARS-COV-2
relative of the titer (%of vehicle)
relative of viral RNA (%of vehicle)
Relative RNA level
Relative subjenomic RNA expression
relative to GAPDH
relative viral genome copy (log10)
relative viral RNA expression
relative virus genome quantities
RNA copies/mL, log 10
RNA copy number (/µL)
SARS-COV-2 E fold change
SARS-COV-2 N fold change
SARS-COV-2 N/GAPDH ddCt
SARS-COV-2 RNA (%)
SARS-COV-2 RNA copy (log10/ml)
SARS-COV-2 viral load (log2 ddCt)
SARS-COV-2 viral load (relative fold change) normalized to RNP/mock
viral gene copies log10 per ml
viral genome copies (log 10)/ ml
viral genome copies (log 10)/µg RNA
viral load (log10 copies per reaction)
viral mRNA (% of GAPDH)
Viral RNA (copies/mL) of
viral titer (PFU/mL)
viral RNA (fold change)
viral RNA basolateral (fold change)
Viral RNA copies/ml
viral RNA load (log10 fold change) normalized to RNP/mock
viral RNA log10 TCID50eq./ ml
Viral titer (TCID50/ ml)
virus genome copy
virus genome copy (%Ctr)
virus genome copy (log10)
virus genome copy /mL (log10)
virus genome copy increased from based line (log10)
Virus titer PFU/mL

TCID50 y-axes

AUC (24-72h)
infectious titer (TCID50/ml)
log virus titre (TCID50/mL)
log10 TCID50 eq./ml
log10 TCID50/ml

TCID50 (log10/ml)
TCID50 /ml (log10)
TCID50 titer/ml
TCID50/mL
titer (TCID50)
Viral titer (TCID50/ml)
**Plaque Assay y-axes**
apical shed SARS-COV-2 (PFU/wash)
FFU/ml 10^5 cells log10
fold change
live virus PFU mL-1
log10 PFU/mL
pfu/mL (%ctr)
PFU/mL (x10^2)
pfu/mL
plaque forming units/mL
Relative viral titer
SARS-COV-2 daily apical shed virus (PFU/wash)
Viral RNA (copies/mL) of viral titer (PFU/mL)
Viral titer (FFU/ml)
Virus titer (pfu/ml)
Virus titer, log10(PFU/mL)

Supplementary Table 6: Sensitivity Analysis: Bayesian Hierarchical Model estimates for ACE2 expression level in the different model systems and respiratory tract: cell cultures, organoids and tissue.

In this model, housekeeping genes were modelled as fixed effects (Compare to Table S10). Note that the PSC: pRT interaction is not reported as there were no data points in this interaction cell, prior has been set to a constant. Residual standard deviation was estimated as σ=0.97±0.19 (sd).

Parameter	Median	Lower 95% CrI	Upper 95% CrI
Housekeeping Gene: OTHER	0,08	-1,01	1,21
Intercept	1,12	-0,07	2,32
Upper Respiratory Tract (URT)	-0,38	-1,61	0,72
3DALI	0,52	-0,81	2,21
3DALI:URT	-0,26	-2,14	1,2
ASC	-0,14	-1,85	1,48
ASC:URT	0,14	-1,73	2,32
COVID Primary	-0,36	-2,55	1,32
COVID Primary:URT	0,23	-1,36	2,14
ex vivo	0,34	-0,85	1,86
ex vivo:URT	-0,77	-2,77	0,66
PSC	0,06	-1,36	1,56

Supplementary Table 7: Sensitivity Analysis: Bayesian Hierarchical Model estimates for TMPRSS2 expression level in the different model systems and respiratory tract: cell cultures, organoids and tissue.

In this model, housekeeping genes were modelled as fixed effects (Compare to Table S11). Note that the PSC: pRT interaction is not reported as there were no data points in this interaction cell, prior was et as a constant. Residual standard deviation was estimated as σ=1.65±0.27 (sd).

Parameter	Median	Lower 95% CrI	Upper 95% CrI
Housekeeping Gene: OTHER	0,1	-1,71	2,01
Intercept	0,88	-1,03	2,85
Upper Respiratory Tract (URT)	0,01	-1,63	1,65
3DALI	-0,19	-2,75	1,94
3DALI:URT	-0,07	-2,52	2,12
ASC	-0,09	-2,53	2,11
ASC:URT	-0,1	-3,11	2,5
COVID Primary	0,06	-2,56	2,84
COVID Primary:URT	-0,07	-2,64	2,23
ex vivo	0,61	-1,06	2,95
ex vivo:URT	0,44	-1,55	3,33
PSC	-0,05	-2,27	2,05

Supplementary Table 8: Organ groups with models and locations

Categorization of the different human organ models and their source material and represented organ compartment in the data set acquired during the pandemic phase. The first column shows the different organ groups (acronyms see table S1). The different model types, cell models, organoids and tissues are shown in the second column. The third column details their specification. And the fourth column details the location that the model is representing according to the information provided in the study. The fifth column shows the total number of the respective model types. The definitions of the model categories can be found in table 1.

Organ	Model	Specification	Localization	#
RT	cell models	2D culture	URT LRT	3
RT	cell models	2D culture	URT	21
RT	cell models	2D culture	LRT	10
RT	cell models	2D culture	ND	1
RT	cell models	3D ALI	URT LRT	1
RT	cell models	3D ALI	URT	15
RT	cell models	3D ALI	LRT	13
RT	organoids	ASC	URT LRT	1
RT	organoids	ASC	URT	3
RT	organoids	ASC	LRT	9

RT	organoids	ASC	ND	1
RT	organoids	PSC	URT	1
RT	organoids	PSC	LRT	7
RT	organoids	PSC	ND	9
RT	tissue	ex vivo	URT LRT	4
RT	tissue	ex vivo	URT	18
RT	tissue	ex vivo	LRT	24
RT	tissue	ex vivo	ND	1
RT	tissue	COVID autopsy	URT LRT	1
RT	tissue	COVID autopsy	LRT	13
CNS	cell models	PSC	cerebral cortex	1
CNS	cell models	PSC	not specified	4
CNS	cell models	PSC	neural cells, neuronal cells	2
CNS	cell models	PSC	neural cells	3
CNS	cell models	PSC	neuronal cells	1
CNS	cell models	PSC	not specified, neuronal cells, neural cells	1
CNS	cell models	PSC	neurovascular unit	1
CNS	cell models	primary	neurovascular unit	1
CNS	cell models	primary	neural cells	1
CNS	organoids	PSC	cerebral cortex	4
CNS	organoids	PSC	not specified	6
CNS	organoids	PSC	neurovascular unit	2
CNS	print	primary	neurovascular unit	1
CNS	tissue	COVID autopsy	cerebral cortex	1
CNS	tissue	ex vivo	cerebral cortex	2
CNS	tissue	ex vivo	not specified	1
CNS	tissue	ex vivo	cerebellum	1
liver	cell models	primary	hepatocytes	2
liver	cell models	primary	liver	1
liver	cell models	primary	hepatocytes, bile duct	1
liver	organoids	PSC	hepatocytes	1
liver	organoids	ASC	hepatocytes	1
liver	organoids	ASC	liver	1
liver	tissue	ex vivo	liver	5
liver	tissue	ex vivo	bile duct	1
liver	tissue	ex vivo	hepatocytes, bile duct	1
liver	chips	ex vivo	liver	1
kidney	cell models	primary	kidney	2
kidney	organoids	PSC	kidney	4
kidney	tissue	ex vivo	kidney	9
kidney	tissue	ex vivo	renal	3
kidney	tissue	COVID autopsy	kidney	1
kidney	chip	primary	kidney	1

skin	cell models	primary	skin	1
skin	organoids	PSC	skin	1
skin	tissue	ex vivo	adipose	3
skin	tissue	ex vivo	skin	2
skin	tissue	COVID autopsy	skin	1
VASC	cell models	primary	capillary	4
VASC	organoids	PSC	capillary	1
GIT	cell models	primary	pancreas	1
GIT	cell models	primary	duodenum,colon	1
GIT	cell models	primary	jejunum	1
GIT	cell models	primary	colon	1
GIT	cell models	PSC	pancreas	1
GIT	organoids	PSC	colon	3
GIT	organoids	PSC	not specified	6
GIT	organoids	ASC	not specified	6
GIT	organoids	ASC	colon	8
GIT	organoids	ASC	ileum	2
GIT	organoids	ASC	stomach	1
GIT	organoids	ASC	duodenum,colon, ileum	1
GIT	tissue	ex vivo	ileum	5
GIT	tissue	ex vivo	colon	3
GIT	tissue	ex vivo	pancreas	2
GIT	tissue	ex vivo	spleen	1
GIT	tissue	ex vivo	esophagus	1
GIT	tissue	ex vivo	not specified	3
GIT	tissue	ex vivo	colon, ileum	1
GIT	tissue	ex vivo	ileum, colon, rectum	1
GIT	tissue	ex vivo	colon, duodenum, ileum, gallbladder	1
GIT	tissue	ex vivo	colon, duodenum, small intestine, gallbladder	1
GIT	tissue	ex vivo	colon, ileum, rectum, stomach,esophagus	1
GIT	tissue	ex vivo	colon, stomach, esophangus, duodenum, gallbladder	1
GIT	tissue	ex vivo	> 5 locations	1
GIT	tissue	COVID autopsy	colon	1
GIT	tissue	COVID autopsy	pancreas	1
heart	cell models	PSC	cardiomyocytes	9
heart	cell models	PSC	cardiac	1
heart	cell models	PSC	cardiomyocytes, stromal	1
heart	cell models	PSC	smooth muscle	1
heart	cell models	primary	artrial, stromal	1
heart	cell models	primary	cardiomyocytes	1

heart	cell models	primary	cardiac	1
heart	cell models	primary	pericytes	1
heart	organoids	PSC	cardiac	1
heart	organoids	PSC	cardiomyocytes	1
heart	tissue	ex vivo	artrial, ventricle	1
heart	tissue	ex vivo	ventricle	2
heart	tissue	ex vivo	cardiac	3
heart	tissue	ex vivo	artrial	2
heart	tissue	ex vivo	cardiomyocytes	4
heart	tissue	ex vivo	pericytes	1
heart	tissue	ex vivo	cardiomyocytes, pericytes, fibroblasts	1
eye	cell models	PSC	retina	1
eye	cell models	primary	cornea	2
eye	cell models	primary	pterygium	1
eye	cell models	primary	ocular	1
eye	cell models	primary	conjunctiva	1
eye	cell models	primary	pterygium, conjunctiva	1
eye	cell models	primary	cornea, limbal, conjunctiva	1
eye	organoids	PSC	eye	1
eye	organoids	PSC	retina	1
eye	tissue	ex vivo	cornea	5
eye	tissue	ex vivo	retina	2
eye	tissue	ex vivo	conjunctiva	2
eye	tissue	ex vivo	ocular, cornea	1
eye	tissue	ex vivo	retina, choroid	1
eye	tissue	ex vivo	pterygium, conjunctiva	1
eye	tissue	ex vivo	cornea, limbal	1
eye	tissue	ex vivo	cornea, limbal, conjunctiva	2
eye	tissue	ex vivo	> 6 localizations	1
eye	tissue	COVID autopsy	conjunctiva, limbal, cornea	1
eye	tissue	COVID autopsy	conjunctiva	1
UGT	cell models	primary	endometric	1
UGT	tissue	ex vivo	placenta	12
UGT	tissue	ex vivo	testicular	2
UGT	tissue	ex vivo	testicular, ovarian	1
UGT	tissue	ex vivo	ovarian,fallopian, breast	1
UGT	tissue	ex vivo	ovarian, oocytes,cumulus	1
UGT	tissue	ex vivo	> 6 localizations	1
UGT	tissue	COVID autopsy	placenta	1

Supplementary Table 9: Explanation of the answer options to describe the differentiation of stem cells

Reviewers were asked to categorize the level of detail in which the stem cell differentiation process was described according to the listed criteria. Multiple choice answers were possible.

Answer option	Explanation for the reviewers
In detail	The study should provide information on e.g., media components, specific type of medium (DMEM High Glucose (4.5 g./l), with L-Glutamine), growth factor, timing, matrices, seeding density, filter size, storage conditions, well sizes, dishes, confluency, etc. (if applicable). Sufficient information should be provided to replicate the differentiation procedure.
Some detail	If only some points of the above-mentioned example are mentioned and the authors do not provide sufficient information to replicate the differentiation procedure
Authors refer to another study	If the authors SOLELY refer to another study (e.g., differentiation was performed according to XXX et al.)
Some detail *AND* Authors refer to another study	If the authors refer to another study AND give additional details (e.g., differentiation was performed as previously described [ref.] Briefly, we cultured…)

Supplementary Table 10: Bayesian Hierarchical Model estimates for ACE2 expression level in the different model systems and respiratory tract: cell cultures, organoids and tissue.

For the ACE2 model, there were 18 publications with 38 data points and an estimate for the residual standard deviation of σ=0.98±0.18 (sd). Note that the PSC: pRT interaction is not reported as there were no data points in this interaction cell. Prior was set to a constant of 0.

Parameter	Median	Lower 95% CrI	Upper 95% CrI
Intercept	1,2	0,11	2,38
Proximal Respiratory Tract (pRT)	-0,37	-1,59	0,72
3DALI	0,37	-1,06	2,06
3DALI:pRT	-0,27	-2,12	1,19
ASC	-0,22	-1,91	1,29
ASC:pRT	0,08	-1,85	2,19
COVID Primary	-0,44	-2,5	1,15
COVID Primary:pRT	0,22	-1,44	2,15
ex vivo	0,41	-0,81	1,98
ex vivo:pRT	-0,89	-3,01	0,59
PSC	0,09	-1,35	1,62

Supplementary Table 11: Bayesian Hierarchical Model estimates for TMRSS2 expression level in the different model systems and respiratory tract: cell cultures, organoids and tissue.

For the TMPRSS2 model, there were 15 publications with 35 data points and an estimate for the residual standard deviation of the intercept σ=1.59±0.25. Note that the PSC: pRT interaction is not reported as there were no data points in this interaction cell, the prior was set to a constant of 0.

Parameter	Median	Lower 95% CrI	Upper 95% CrI
Intercept	1,02	-0,64	3,46
Proximal Respiratory Tract (pRT)	-0,07	-1,54	1,35
3DALI	-0,41	-2,75	1,34
3DALI:pRT	-0,1	-2,27	1,89
ASC	-0,24	-2,31	1,51
ASC:pRT	-0,23	-3,01	1,94
COVID Primary	0,13	-1,68	2,06
COVID Primary:pRT	-0,05	-2,36	2,22
ex vivo	0,66	-0,82	2,7
ex vivo:pRT	0,08	-1,96	2,32
PSC	-0,08	-2,22	1,91

Supplementary Table 12: Overview of the different virus strains

The virus types to infect the models we extracted according to the provided information in the studies. Of the 136 studies that performed infections, 56 different SARS-CoV-2 virus types were utilized.

Virusnames	Total number
USA-WA1/2020	44
USA/CA-UCSF-0001C/2020	1
USA-WA1/2020,icSARS-CoV-2mNG	1
BetaCoV/Munich/BavPat1/2020	9
BetaCoV/Korea/KCDC03/2020	1
BetaCoV/Hong Kong/VM20001061/2020	2
BetaCov/Netherlands/01/NL/2020	5
BetaCoV/Australia/VIC01/2020	1
BetaCoV/Australia/VIC18440/2021	1
BetaCoV/Germany/BavPat1/2020	3
BetaCoV/France/IDF0372/2020	2
SARS-CoV-2/human/HKG/HKU-001a/2020	4
SARS-CoV-2/human/USA/CA-UCSF-0001C/2020	1
SARS-CoV-2/human/Liverpool/REMRQ0001/2020	2
hCoV-19/Hong Kong/3268-P2/2020	1

hCoV-19/Hong Kong/2161-P5/2020	1
hCoV-19/Hong Kong/4257-P2/2021	1
hCoV-19/Hong Kong/VOC0013P2D5/2021	1
hCoV-19/Hong Kong/VOC0195-P2/2021	1
hCoV-19/Japan/TY7-503/2021	2
hCoV-19/USA/CA-SU-15_S02/2021	1
SARS-CoV-2/human/SWE/01/2020	2
SARS-CoV-2/1/Human/2020/Frankfurt	1
SARS-CoV-2/SH01/human/2020/CHN	2
SARS-CoV-2 WK-521	1
SARSCoV-2 isolate HH-1	1
SARS-CoV-2 isolate Wuerzburg	1
SARS-CoV-2/Wuhan-1/2020	1
SARS-CoV-2/Munchen1.1/2020/929	1
SARS-CoV-2 NRW-42	1
SARS-CoV-2 SZTH-003	1
SARS-CoV-2 SZ454	1
SARS-CoV-2 (WIV04)	2
SARS-CoV-2-FFM1	1
SARS-CoV-2/human/Switzerland/IMV5/2020	1
hCoV-19/South Africa/KRISP-K005325/2020	1
B .1.1.617.2 (Delta)	1
B .1.1.529 Omicron UK isolate47	1
Omicron South Africa	1
Omicron isolate (BA.1 lineage, strain TY38-873)	1
UW-001/Human/2020/Wisconsin (UW-001)	1
2019-nCoV/Japan/TY/WK-521/2020	1
JPN/Kanagawa/KUH003	1
QK002	1
TY7-501	1
hCoV-19/Norway/Bergen-01/2020	1
hCoV-19/Norway/Trondheim-S15/2020	1
hCoV-19/England/204661721/2020	1
hCoV-19/England/204501206/2020	1
hCoV-19/England/204501194/2020	1
hCoV-19/England/204820464/2020	1
hCoV-19/Hong Kong/WHV-HK61-P3/2020	1
hCoV-19/USA/PHC658/2021	1
hCoV-19/Australia/QLD02/2020	1
2019-nCoV/Italy-INMI1	1
REMRQ001; B.1.1.7; B.1.617.2	1

Supplementary Table 13: Results of meta-analysis on viral replication in respiratory tract models

Bayesian non-linear hierarchical model using a reparametrized Gompertz function to model viral load increase. Note that parameter k (maximum slope) for each model is parametrized as effect and not as contrast. The analysis includes were 13 publications with 130 data points and an estimate for the

residual standard deviation of σ=0.37±0.03 (sd). Pairwise comparisons between k factors did not indicate differences between them with Bayes factors for all comparison ~0.4.

Parameter	Median	Lower 95% CrI	Upper 95% CrI
k ex vivo	0,1	0,07	0,47
k organoids	0,09	0,07	0,28
k tissue	0,11	0,07	0,24
lag	1,6	-2,45	5,67
y0	-1,23	-1,44	-0,81
y_max	2,27	1	4,11

Supplementary Table 14: Heterogeneity Measure from Bayesian Hierarchical Model for PCR ACE2

Estimates extracted via draws from posterior

Column1	Column2
Parameter	Estimate [95% CrI]
Tau (τ): DOI_	0.958 [0.109, 1.819]
Tau (τ): housekeeping_gene_	0.485 [0.02, 1.915]
Total I² (Heterogeneity)	61.7% [11.9%, 87.5%]

Supplementary Table 15: Heterogeneity Measure from Bayesian Hierarchical Model for PCR TMPRSS2

Estimates extracted via draws from posterior

Column1	Column2
Parameter	Estimate [95% CrI]
Tau (τ): DOI_	0.48 [0.02, 2.274]
Tau (τ): housekeeping_gene_	2.54 [0.342, 5.476]
Total I² (Heterogeneity)	74.8% [41.4%, 93.2%]

Supplementary Table 16: Heterogeneity Measure from Bayesian Hierarchical Model for viral infection

Estimates extracted via draws from posterior

Column1	Column2
Parameter	Estimate [95% CrI]
Tau (τ): PDF__y0	0.09 [0.004, 0.312]
Tau (τ): PDF__ymax	0.352 [0.119, 0.899]
Tau (τ): PDF__k	0.07 [0.004, 1.206]

Tau (τ): PDF__lag	11.695 [3.041, 21.705]
Total I² (Heterogeneity)	99.9% [98.7%, 100%]

Supplementary Methods 1: Bayesian Modelling of Host Factor Expression in Respiratory Tissues

To perform meta-analyses on the host factor expression in different model systems, we extracted quantitative data points from qPCRs plots. We extracted 122 data points on the host factor expression levels from 74 figures of 32 studies. In over half of the studies (n=17), expression levels were normalized to one category within the study (e.g. treatment normalized to mean of control). As these values are not comparable between studies, we included only studies that reported relative expression levels to a housekeeping gene.

We were unable to consistently extract measures of spread (standard error of the mean or standard deviation) and/or sample sizes from studies, as these were frequently not reported or specified (n=13 out of 38 (ACE2) and n=7 out of 34 (TMPRSS2) data points) (Fig. S1). We present here a model where we did not assign a weight to each study in a Bayesian hierarchical model.

The meta-analyses were conducted using the *brms* package, version 2.22.0 in R 4.4.1. for model fitting and inference. We applied Bayesian modelling in three meta-analyses. The first two meta-analyses investigate host factor expression for ACE2 and TMPRSS2 for measurements within the pRT and dRT and each model type.

Housekeeping genes differed between studies included in the final analysis (GAPDH: n=10, Cytokeratin: n=1, HPRT1: n= 1; PPIA: n=2; TBP: n=1 studies). To account for variation in base levels between housekeeping genes, we included a random effect on the intercept for each housekeeping gene when modeling ACE2 and TMPRSS2. Clusters were small for all housekeeping genes except GAPDH (Fig. S2). To ensure, results were not influenced by high uncertainties within these clusters, we also included a sensitivity analysis where we collapsed all housekeeping genes that were not GAPDH in one category and entered it as a fixed effect (Tables S6 & S7).

Our initial analysis plan was a traditional meta-analysis that considers mean values, measures of variance, and sample sizes in each study to assess effects. For the reasons outlined above, this was not possible, and as a result, all meta-analysis findings are highly exploratory and should be interpreted with caution.

Bayesian Hierarchical Modelling

Bayesian hierarchical models were employed to analyse the expression of ACE2 and TMPRSS2 across different respiratory tract models (cell cultures, organoids, tissue), locations (pRT, dRT), and specifications (e.g., cell types, species). The *brms* package, *version 2.22.0* in R was utilized for model fitting and inference (RRID:SCR_023862**)** and fit in R 4.4.1.

Model Structure: The models were specified as follows:

Outcome: Expression levels.

Fixed Effects: Location (pRT vs. dRT), specification (categorical), and their interaction.

Random Effects: Varying intercepts for each study and house-keeping gene to account for between-study heterogeneity.

Priors: Weakly informative priors were assigned to all model parameters, allowing the data to drive the inference.

$$\begin{aligned} \text{Intercept} &\sim \text{Student-}t(1,0,1) \quad \text{with lower bound } 0 \\ b &\sim \text{Student-}t(1,0,1) \\ \sigma &\sim \text{Cauchy}(0,1) \end{aligned}$$

Model Fitting: Models were fit using Markov Chain Monte Carlo (MCMC) methods, with four chains and 10000 iterations (plus 10000 warm-up iterations) to ensure convergence. Convergence was checked visually and Gelman-Rubin statistics (R-hat<1.05, lowest effective sample size was Bulk>10000; Tail>2000).

Inference: Posterior distributions of model parameters were used to estimate effect sizes, credible intervals, and to test hypotheses regarding differences in expression between locations and across specifications.

Supplementary Methods 2: Bayesian Modelling of Infection Curves Using Gompertz Function

Gompertz Growth Model

The Gompertz function was used to model the growth of viral load over time. The Gompertz function is a sigmoid curve commonly used to describe biological growth processes with an initial lag phase, followed by an exponential increase, and eventually reaching an asymptote[1].

Gompertz Equation

$$y(t) = y_0 + (y_{max} - y_0) \cdot exp(-exp(k \cdot \frac{lag - t}{y_{max} - y_0} + 1))$$

where:

- y(t): Viral load at time t
- y_0: Initial viral load
- y_{max}: Maximum viral load (asymptote)
- k: Maximum growth rate
- lag: Time of maximum growth rate (inflection point)

Bayesian Hierarchical Modelling

Bayesian nonlinear hierarchical models were employed to estimate the parameters of the Gompertz function for each experimental model (cell lines, primary cells, etc.). The brms package in R was utilized for model fitting and inference.

Model Structure

Outcome: Within study Z-transformed viral load.

Linear Fixed Effects: Experimental model as a factorial effect on the maximum growth rate.

Random Effects: Varying intercepts for each study to account for between-study heterogeneity for each model parameter.

Priors: Weakly informative priors were assigned to all model parameters.

$$\begin{aligned}
\mathrm{y0} &\sim \mathcal{N}(-1, 2) \\
\mathrm{ymax} &\sim \mathcal{N}(1, 2), \text{with lower bound} = 0.001 \\
\mathrm{k} &\sim \mathcal{N}(0, 2) \\
\mathrm{lag} &\sim \mathcal{N}(0, 2)
\end{aligned}$$

Model Fitting: Models were fit using Markov Chain Monte Carlo (MCMC) methods, with four chains and 20000 iterations to ensure convergence. Convergence was checked visually and Gelman-Rubin statistics (R-hat<1.05, Effective Samples: Bulk> 800, Tails>500).

Inference: Posterior distributions of model parameters were used to estimate Gompertz parameters, credible intervals, and to test hypotheses regarding differences in growth dynamics between experimental models.

Supplementary Methods 3: Protocol Development and Preregistration

This SR was preregistered on the International Prospective Register of Systematic Reviews (PROSPERO, CRD42021250999) on June 9, 2021. Due to the heterogeneity of experimental designs and reporting in the investigated studies we had to adapt our protocol. Deviations from our registered protocol were reported on PROSPERO during an update on May 23, 2022. The data extraction protocol focused on methodological procedures to generate human 2D/3D models, technical details of various assays, the reported results and the presentation of the results.

Supplementary Methods 4: Systematic Literature Search Strategy and Study Selection

The systematic literature search was performed on PubMed and the WHO COVID-19 research database. The WHO database enabled a targeted search on 32 bibliographic and grey literature sources. The foundational search concepts were systematically categorized into six key domains: Disease, Model System, Host Factors, Organism, Language, and Publication Type.

The processing of the identified studies was divided into four stages:
Stage 1 – Deduplication in Endnote
Stage 2 – Title and abstract screen on RAYYAN to exclude non-primary research articles
Stage 3 – pdf retrieval and merge with supplemental files
Stage 4 – Full text screen and labeling: A decision about eligibility of the study for data abstraction was made independently by two reviewers based on *a priori* defined inclusion and exclusion criteria (table S2).

The studies were categorized and labeled accordingly: Respiratory Tract (RT), Gastrointestinal Tract (GIT), Central Nervous System (CNS), Heart, Urogenital Tract (UGT), Kidney, Liver, Skin/Adipose tissue, Vasculature (VASC). Due to the high complexity of RNA sequencing techniques, a small team of RNA sequencing domain experts reviewed a separate set of studies reporting exclusively RNA sequencing experiments (labeled as SEQ).

Supplementary Methods 5: Training

The learnings from the intense training sessions led to the elaborate expansions of explanation boxes in the data extraction forms in the NUMBAT tool to accommodate the heterogeneity of reporting *in vitro* studies. These explanation boxes on each question were experienced as very helpful by the reviewers and exceeded the usability of a reviewer handbook.

Supplementary Methods 6: Data Collection, Data Handling and Analysis

We developed five separate data extraction forms (Study Eligibility: https://osf.io/xay9b/files/acukv; Hostfactor:https://osf.io/6b2pc;Infection:https://osf.io/5ju4y; Immune Response: https://osf.io/745n6 ; RNA SEQ: https://osf.io/xay9b/files/ncdyj) with different answer settings of single and multiple choice, free text and table response formats. Free text options were used for comments or implemented with self-learning drop down strings. The table format was used for data extractions when default answer options were not possible, e.g., for cell types, values or units. The data extraction form for the pandemic dataset contained 184 questions (104 questions on reporting quality and experimental design, 80 on data point extractions). For the post-pandemic data set we streamlined the question catalog to 23 questions on model complexity and reporting quality, based on the main outcomes of the analysis of the pandemic data.

Supplementary Methods 7: PRISMA checklist

Section and Topic	Item #	Checklist item	Location where item is reported
**TITLE: Low Reporting Quality Limits the Contribution of Human Organ Models to COVID-19 Research: A Systematic Review**			
Title	1	Identify the report as a systematic review.	p.1
**ABSTRACT**			
Abstract	2	See the PRISMA 2020 for Abstracts checklist.	p.2
**INTRODUCTION**			
Rationale	3	Describe the rationale for the review in the context of existing knowledge.	pp.2 & 4
Objectives	4	Provide an explicit statement of the objective(s) or question(s) the review addresses.	pp. 4 & 5
**METHODS**			
Eligibility criteria	5	Specify the inclusion and exclusion criteria for the review and how studies were grouped for the syntheses.	S table 2 & 3<br>S method 4
Information sources	6	Specify all databases, registers, websites, organisations, reference lists and other sources searched or consulted to identify studies. Specify the date when each source was last searched or consulted.	p. 6<br>S method 4<br>https://osf.io/xay9b/files/r7pgn
Search	7	Present the full search strategies for all	p. 6

strategy		databases, registers and websites, including any filters and limits used.	S table 3<br>https://osf.io/xay9b/files/bk5cg
Selection process	8	Specify the methods used to decide whether a study met the inclusion criteria of the review, including how many reviewers screened each record and each report retrieved, whether they worked independently, and if applicable, details of automation tools used in the process.	pp. 6 & 7<br>S method 5 & 6
Data collection process	9	Specify the methods used to collect data from reports, including how many reviewers collected data from each report, whether they worked independently, any processes for obtaining or confirming data from study investigators, and if applicable, details of automation tools used in the process.	p. 7<br>S method 6<br>https://osf.io/xay9b/files/osfstorage
Data items	10a	List and define all outcomes for which data were sought. Specify whether all results that were compatible with each outcome domain in each study were sought (e.g. for all measures, time points, analyses), and if not, the methods used to decide which results to collect.	p. 7<br>S method 1 & 2<br>S Figure 1<br>https://osf.io/xay9b/files/tj2me
	10b	List and define all other variables for which data were sought (e.g. participant and intervention characteristics, funding sources). Describe any assumptions made about any missing or unclear information.	Table 1<br>p. 7<br>https://osf.io/xay9b/files/osfstorage
Study risk of bias assessment	11	Specify the methods used to assess risk of bias in the included studies, including details of the tool(s) used, how many reviewers assessed each study and whether they worked independently, and if applicable, details of automation tools used in the process.	p. 2
Effect measures	12	Specify for each outcome the effect measure(s) (e.g. risk ratio, mean difference) used in the synthesis or presentation of results.	p. 7
Synthesis methods	13a	Describe the processes used to decide which studies were eligible for each synthesis (e.g. tabulating the study intervention characteristics and comparing against the planned groups for each synthesis (item #5)).	https://osf.io/xay9b/files/tj2me
	13b	Describe any methods required to prepare the data for presentation or synthesis, such as handling of missing summary statistics, or data conversions.	S Figure 1
	13c	Describe any methods used to tabulate or visually display results of individual studies and syntheses.	p. 7
	13d	Describe any methods used to synthesize results and provide a rationale for the choice(s). If meta-analysis was performed, describe the model(s), method(s) to identify the presence and extent of statistical heterogeneity, and software package(s) used.	pp.9-10<br>S method 1 & 2<br>(S table 6, 7, 10, 11)
	13e	Describe any methods used to explore possible causes of heterogeneity among	p.7<br>S method 1 & 2

		study results (e.g. subgroup analysis, meta-regression).	(S tables 14-16)
	13f	Describe any sensitivity analyses conducted to assess robustness of the synthesized results.	S table 6 & 7
Reporting bias assessment	14	Describe any methods used to assess risk of bias due to missing results in a synthesis (arising from reporting biases).	p. 2
Certainty assessment	15	Describe any methods used to assess certainty (or confidence) in the body of evidence for an outcome.	p. 7<br>S method 1
**RESULTS**			
Study selection	16a	Describe the results of the search and selection process, from the number of records identified in the search to the number of studies included in the review, ideally using a flow diagram.	Fig. 1
	16b	Cite studies that might appear to meet the inclusion criteria, but which were excluded, and explain why they were excluded.	Fig. 1<br>https://numbat.bgcarlisle.com/organs/
Study characteristics	17	Cite each included study and present its characteristics.	Fig. 2<br>https://numbat.bgcarlisle.com/organs/
Risk of bias in studies	18	Present assessments of risk of bias for each included study.	p. 17<br>(S table 6 & 7)
Results of individual studies	19	For all outcomes, present, for each study: (a) summary statistics for each group (where appropriate) and (b) an effect estimate and its precision (e.g. confidence/credible interval), ideally using structured tables or plots.	pp. 8- 15<br>Fig. 2-5
Results of syntheses	20a	For each synthesis, briefly summarise the characteristics and risk of bias among contributing studies.	pp. 8- 15<br>S figure 3- 9
	20b	Present results of all statistical syntheses conducted. If meta-analysis was done, present for each the summary estimate and its precision (e.g. confidence/credible interval) and measures of statistical heterogeneity. If comparing groups, describe the direction of the effect.	pp. 11 & 12, 13 & 14<br>S table 10 & 11 & 13<br>S figure 2 & 10 & 11
	20c	Present results of all investigations of possible causes of heterogeneity among study results.	S table 14-16
	20d	Present results of all sensitivity analyses conducted to assess the robustness of the synthesized results.	S table 6 & 7<br>S figure 2
Reporting biases	21	Present assessments of risk of bias due to missing results (arising from reporting biases) for each synthesis assessed.	p. 17
Certainty of evidence	22	Present assessments of certainty (or confidence) in the body of evidence for each outcome assessed.	S table 10, 11, 13
**DISCUSSION**			
Discussion	23a	Provide a general interpretation of the results in the context of other evidence.	pp. 2, 16- 18

	23b	Discuss any limitations of the evidence included in the review.	p. 16
	23c	Discuss any limitations of the review processes used.	p.16
	23d	Discuss implications of the results for practice, policy, and future research.	p. 16- 19
**OTHER INFORMATION**			
Registration and protocol	24a	Provide registration information for the review, including register name and registration number, or state that the review was not registered.	S method 3<br>PROSPERO, CRD42021250999
	24b	Indicate where the review protocol can be accessed, or state that a protocol was not prepared.	S method 3<br>PROSPERO<br>CRD42021250999<br>https://osf.io/xay9b/files/cx4hr
	24c	Describe and explain any amendments to information provided at registration or in the protocol.	S method 3
Support	25	Describe sources of financial or non-financial support for the review, and the role of the funders or sponsors in the review.	pp. 2 & 20
Competing interests	26	Declare any competing interests of review authors.	p. 19
Availability of data, code and other materials	27	Report which of the following are publicly available and where they can be found: template data collection forms; data extracted from included studies; data used for all analyses; analytic code; any other materials used in the review.	https://osf.io/xay9b/files/osfstorage

Supplementary Figure 1: Extraction of observations for meta-analyses for host factors ACE2 and TMPRSS2

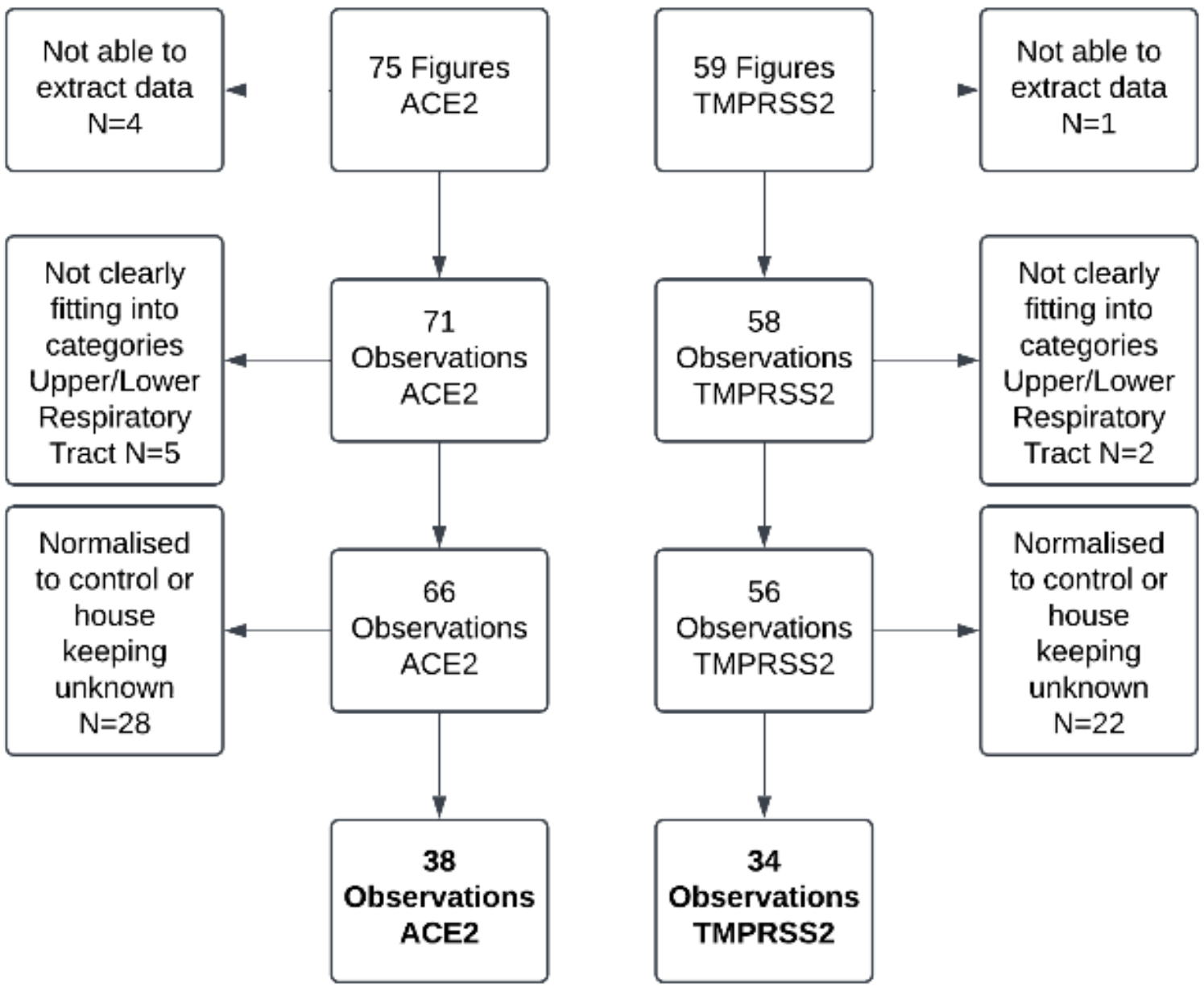

Supplementary Fig. 1: Flowchart exclusion of data points from data set to final analysis data set.

Supplementary Figure 2: Posterior predictive plots for meta-analyses on host factors for ACE2 (upper panel) and TMPRSS2 (lower panel).

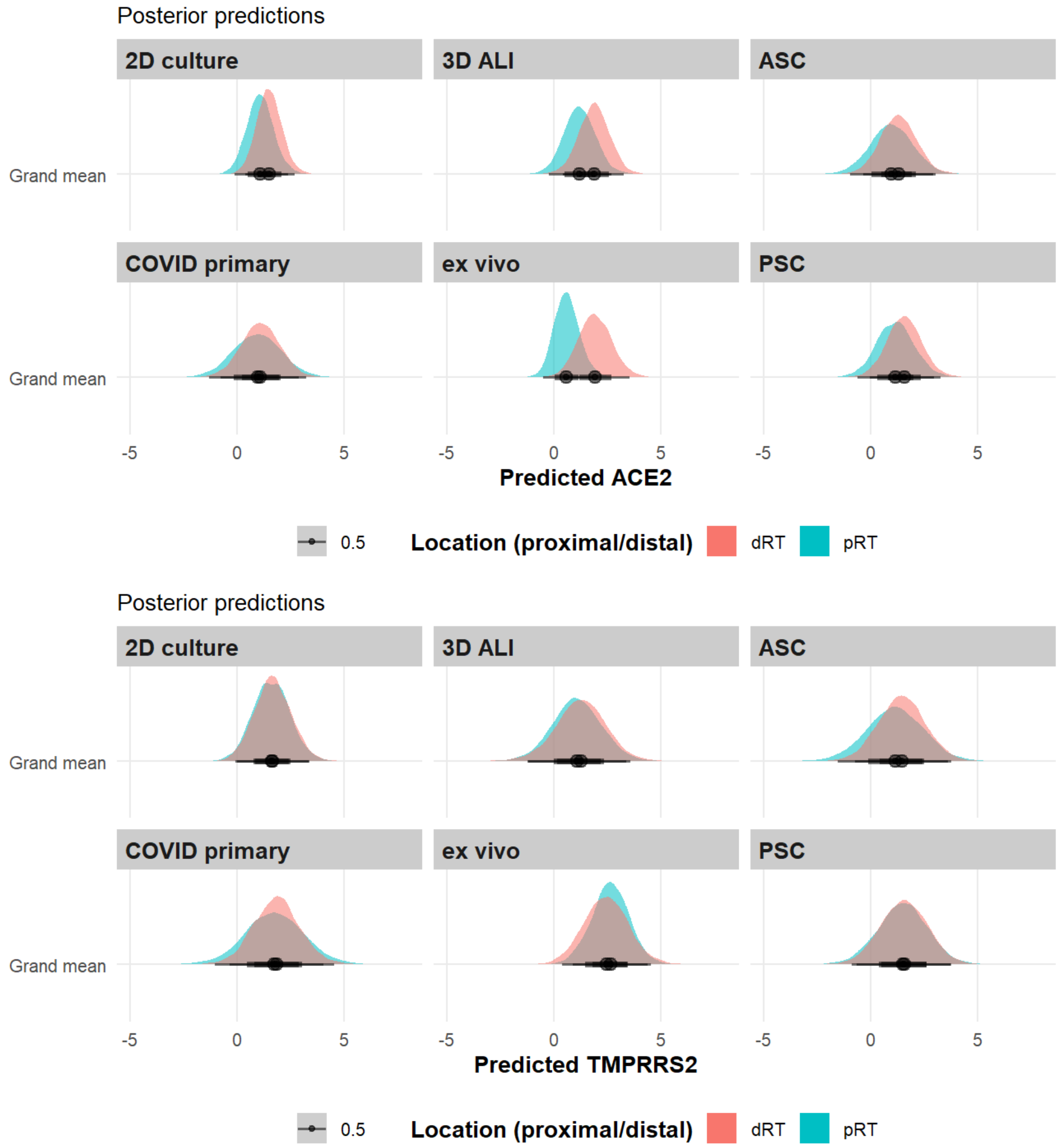

Supplementary Fig. 2: Posterior predictive plots for ACE2 (upper panel) and TMPRRS2 (lower panel). Depicted are posterior distributions for the variables of interest in in our meta-analyses.

Sensitivity Analysis: We conducted a sensitivity analysis by entering the housekeeping gene as a fixed factor. We collapsed all non GAPDH genes in one category to test whether high uncertainty in cluster estimates in the random effects model were influencing covariate estimates. There were no striking differences between the two analyses (Table S10, S11).

Supplementary Figure 3: Metadata and Quality Control in RNA Sequencing experiments

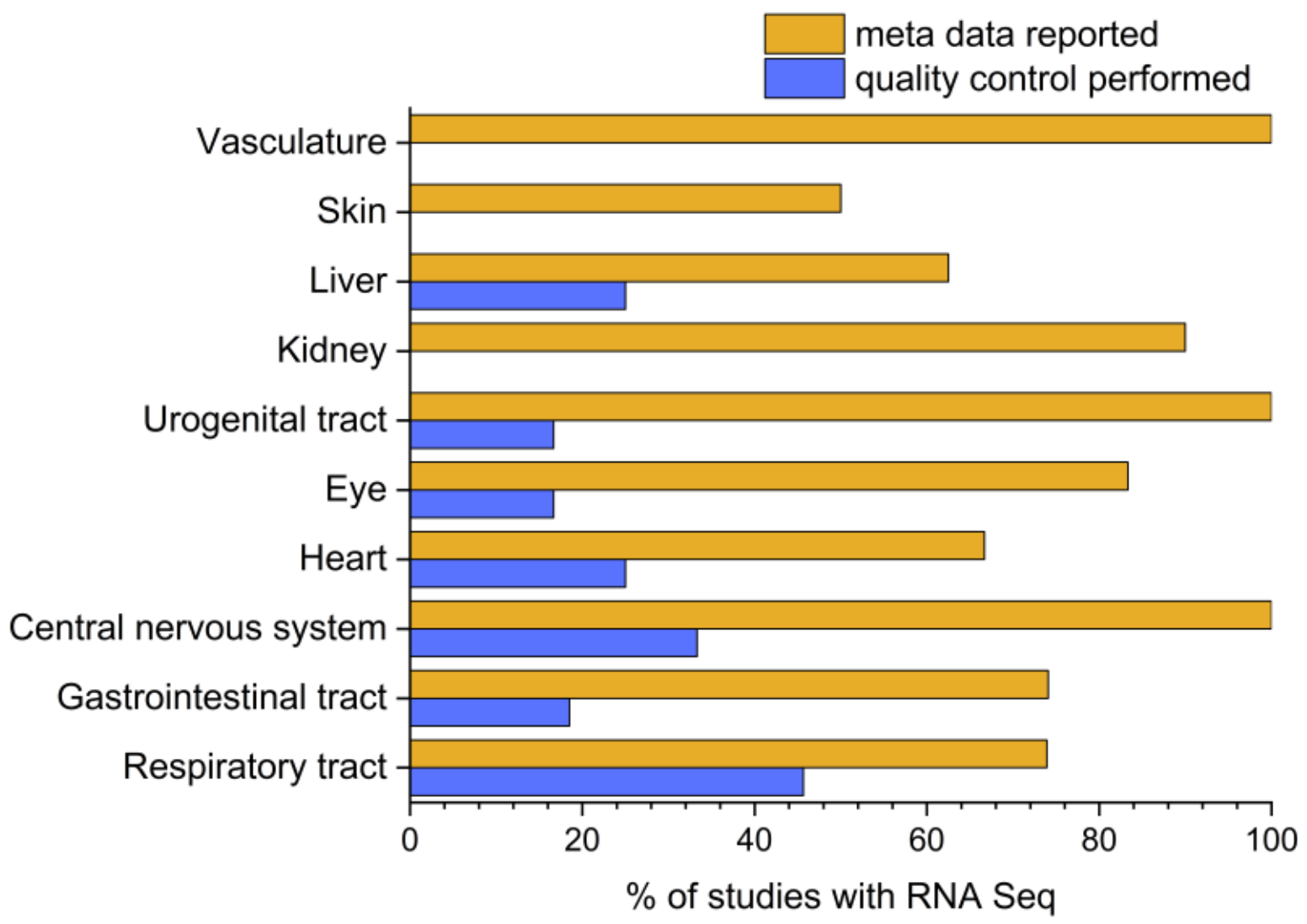

Supplementary Fig. 3: Summary of the percentage of studies with reported metadata for measured patient samples and reported quality controls.

Supplementary Figure 4: Plaque Assay Images

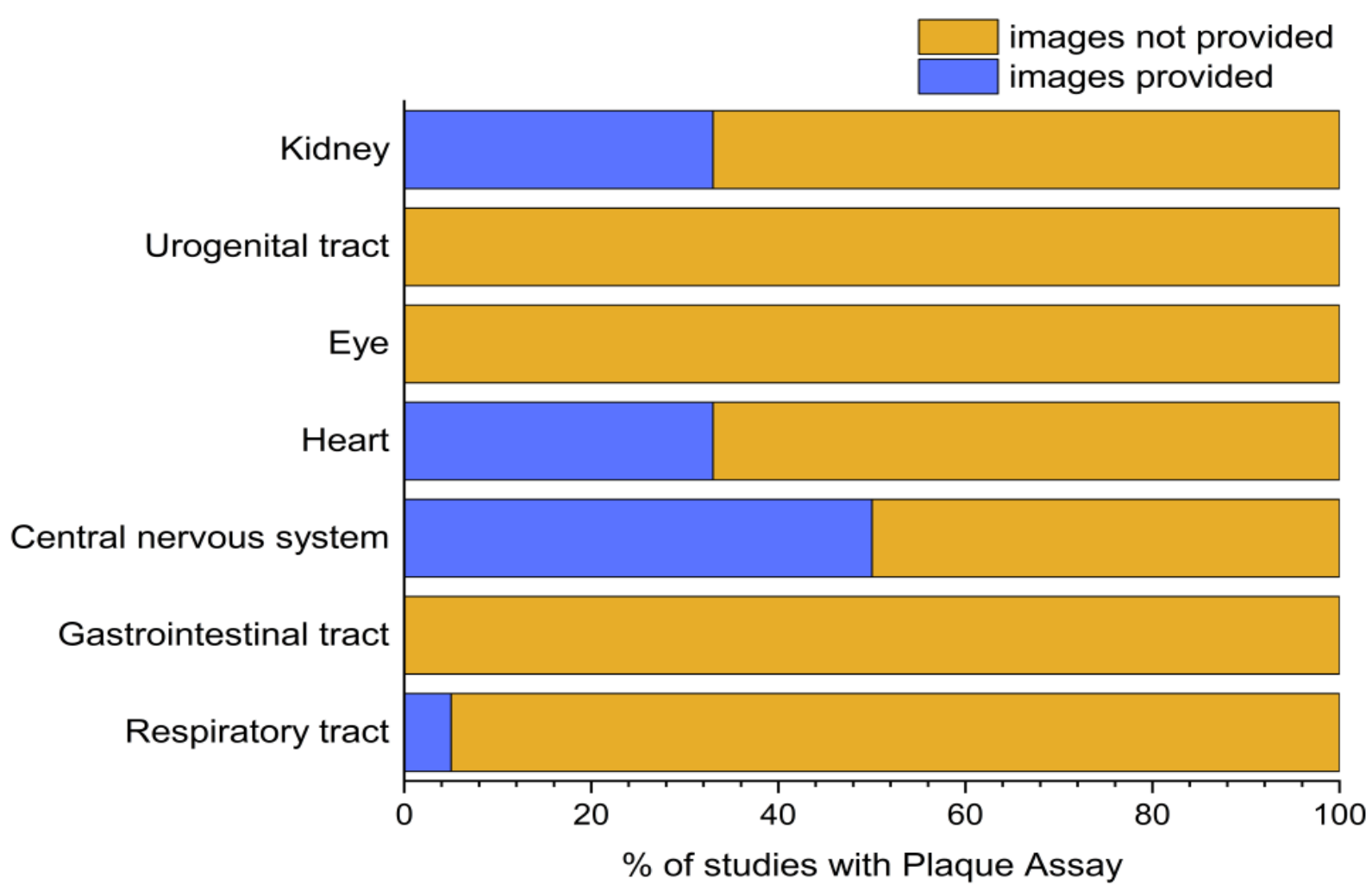

Supplementary Fig. 4: We extracted if studies presented images of the Plaques/dishes in case they performed a Plaque Assay. While 50% of the CNS studies did provide information on the Plaque assay images, most other studies did not or to a small extent.

Supplementary Figure 5: Primer Sequence

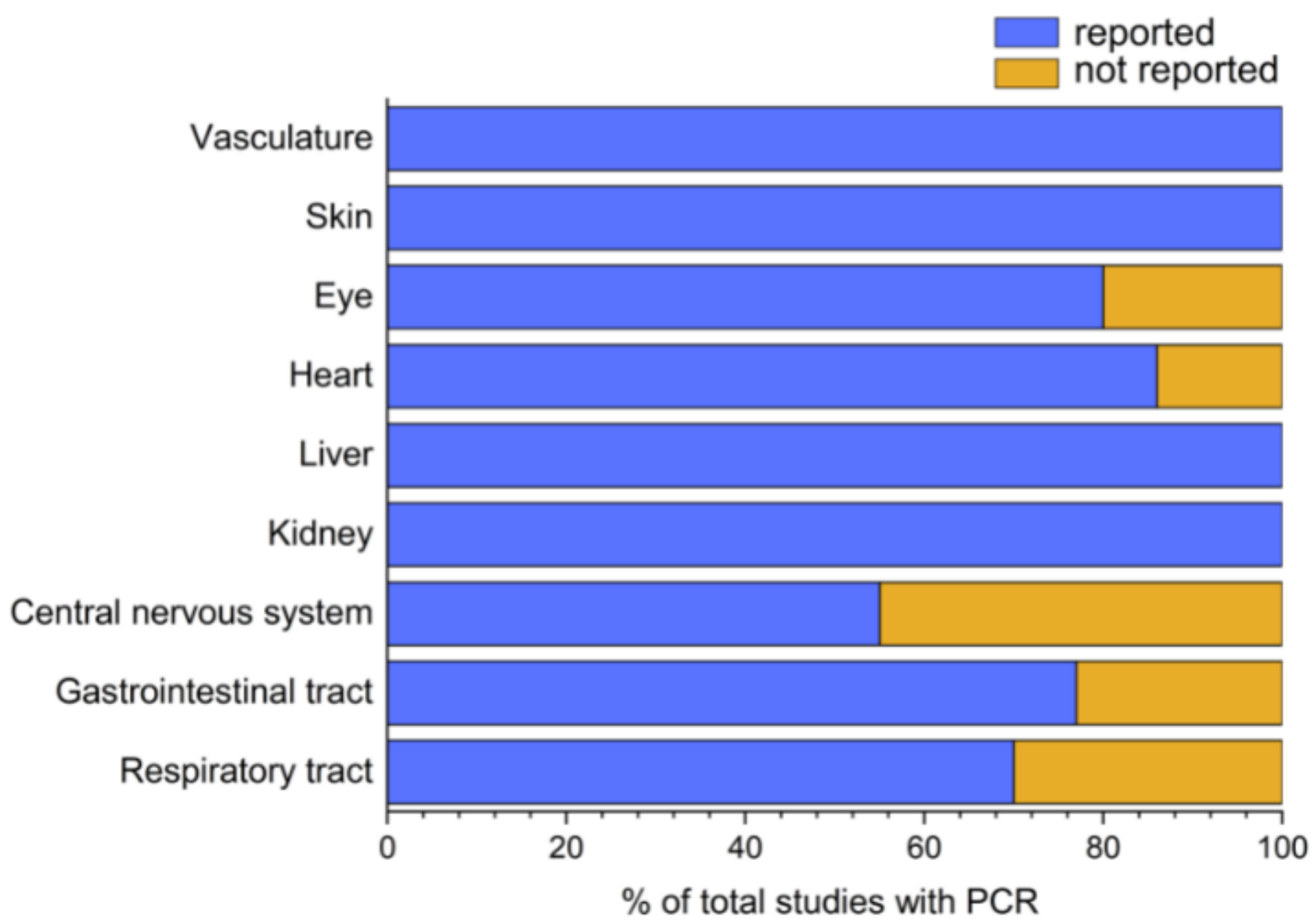

Supplementary Fig. 5: We extracted if the studies provided the primer sequences in case, they performed a qPCR. Independent of organ compartment at least 50 % of studies reported the sequences of the used primers.

Supplementary Figure 6: Reported details on culture conditions for models during the pandemic (A) and post-pandemic period (B)

A

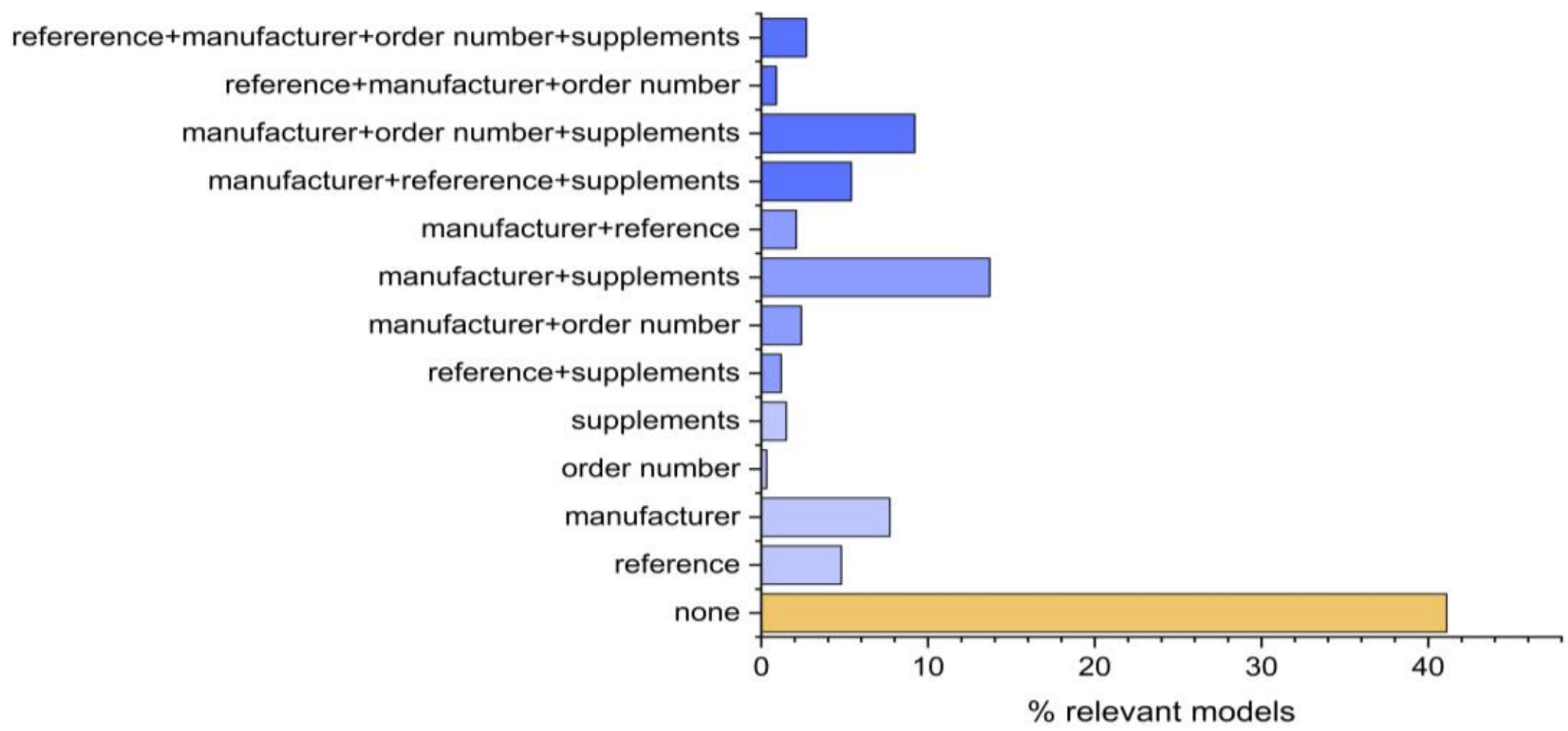

B

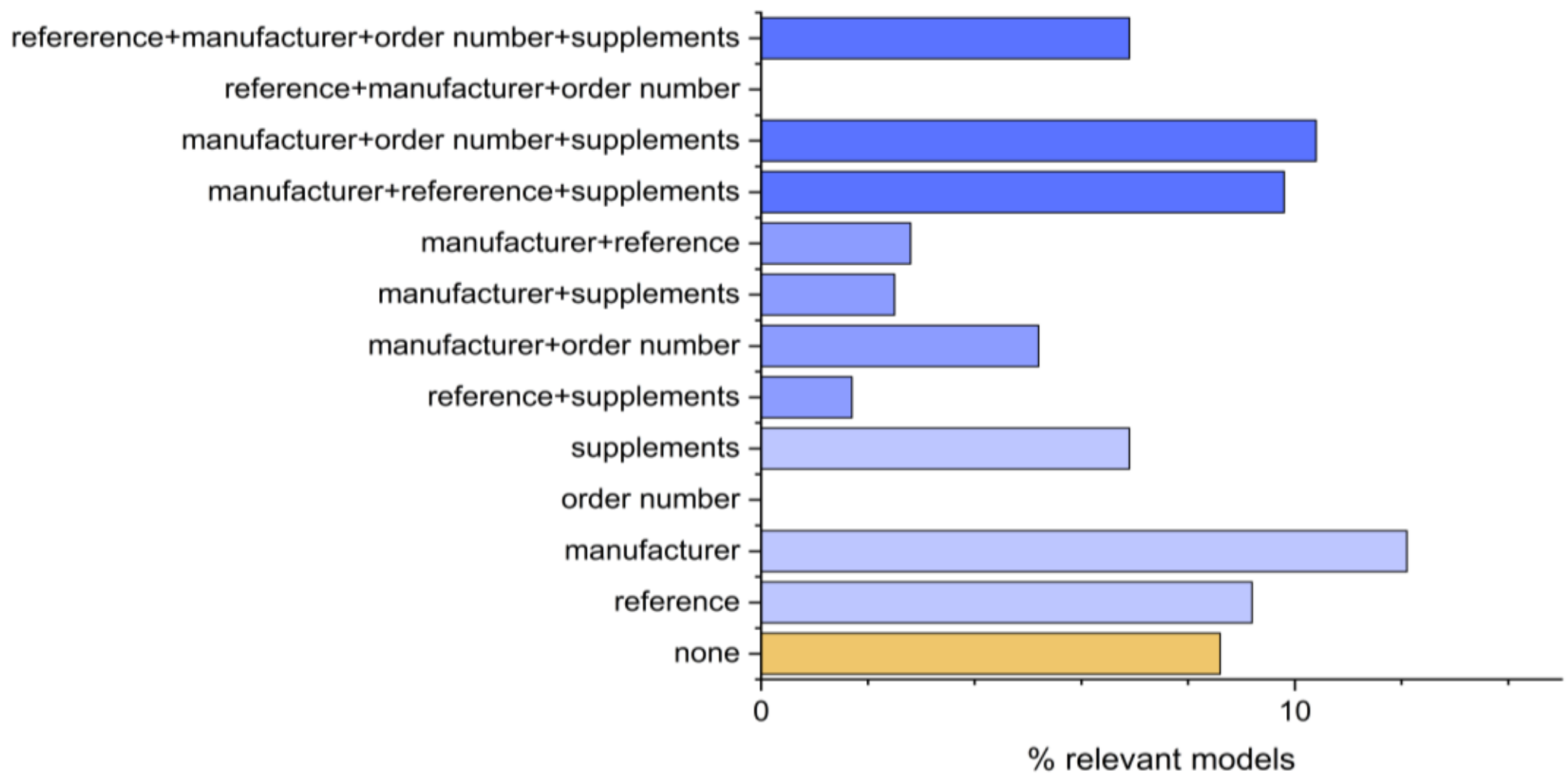

Supplementalry Fig. 6: The details of the culture conditions reported for relevant models (i.e. models kept in culture) were investigated. Predefined information included manufacturer, supplements, reference, and order number.

Supplementary Figure 7: Reported details on stem cell differentiation during the pandemic (A) and post-pandemic period (B)

A

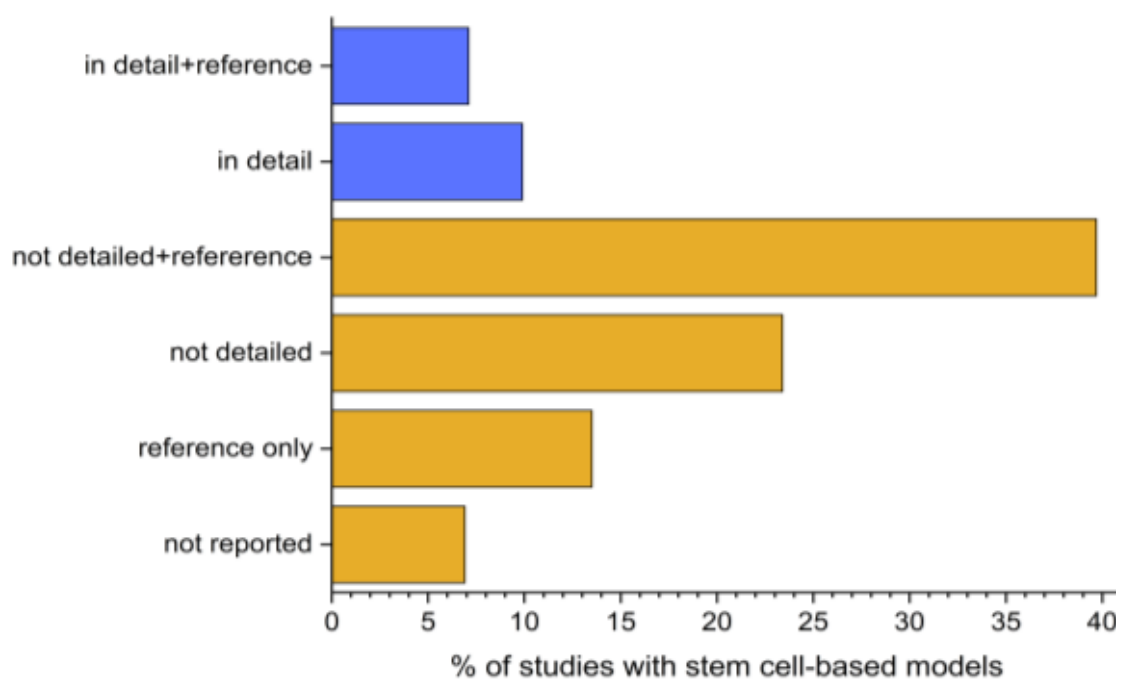

B

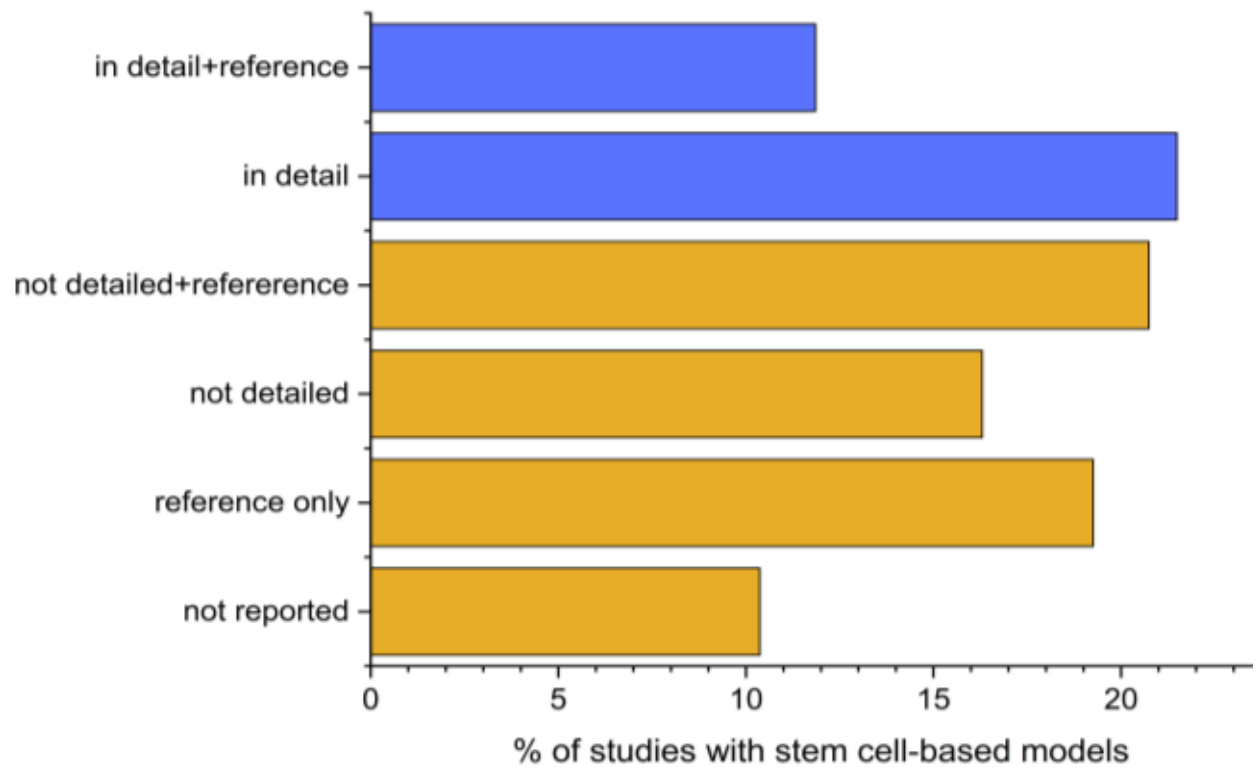

Supplementary Fig. 7: For stem cell-based models, the detail of the reported differentiation process was extracted, with categories: detailed, reference provided, not detailed, and not reported

Supplementary Figure 8: Reported metadata for patient derived material during the pandemic (A) and post-pandemic period (B)

A B

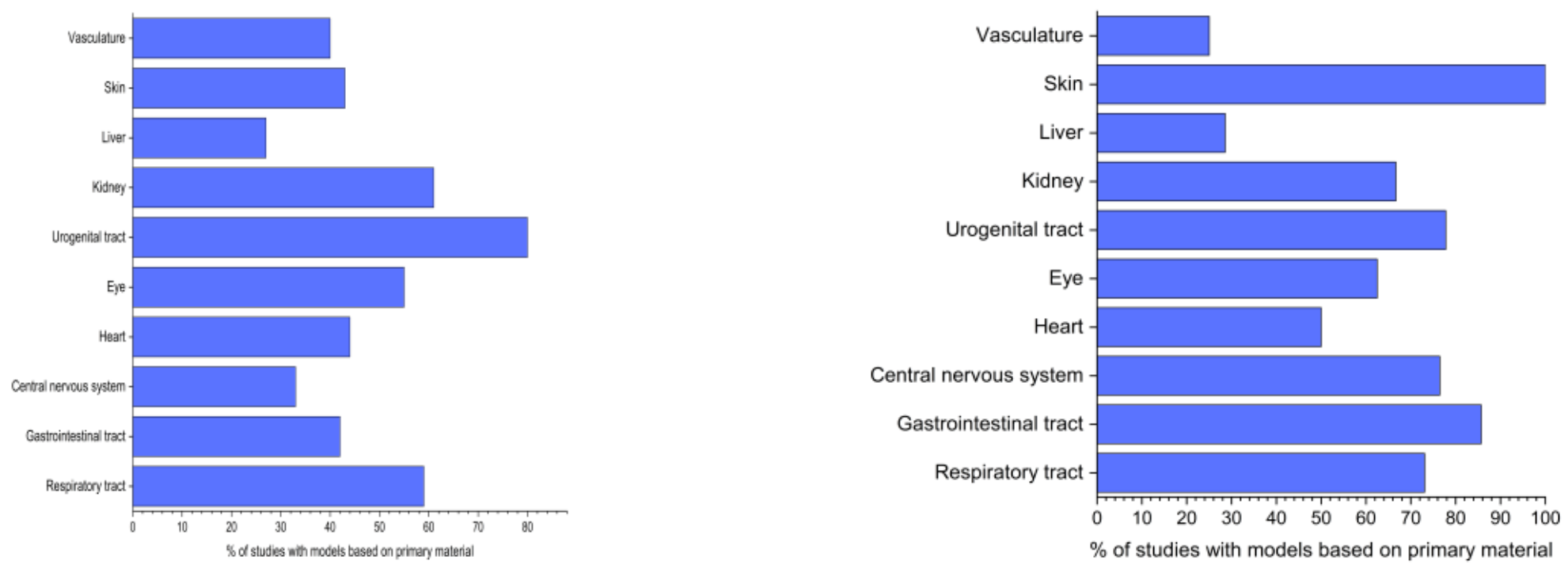

Supplementary Fig. 8: For models based on primary material, it was extracted whether any metadata of the donor (biological sex, age, health status) were presented in the studies.

Supplementary Figure 9: Number of investigated areas per study during the pandemic (A) and post-pandemic period (B)

A B

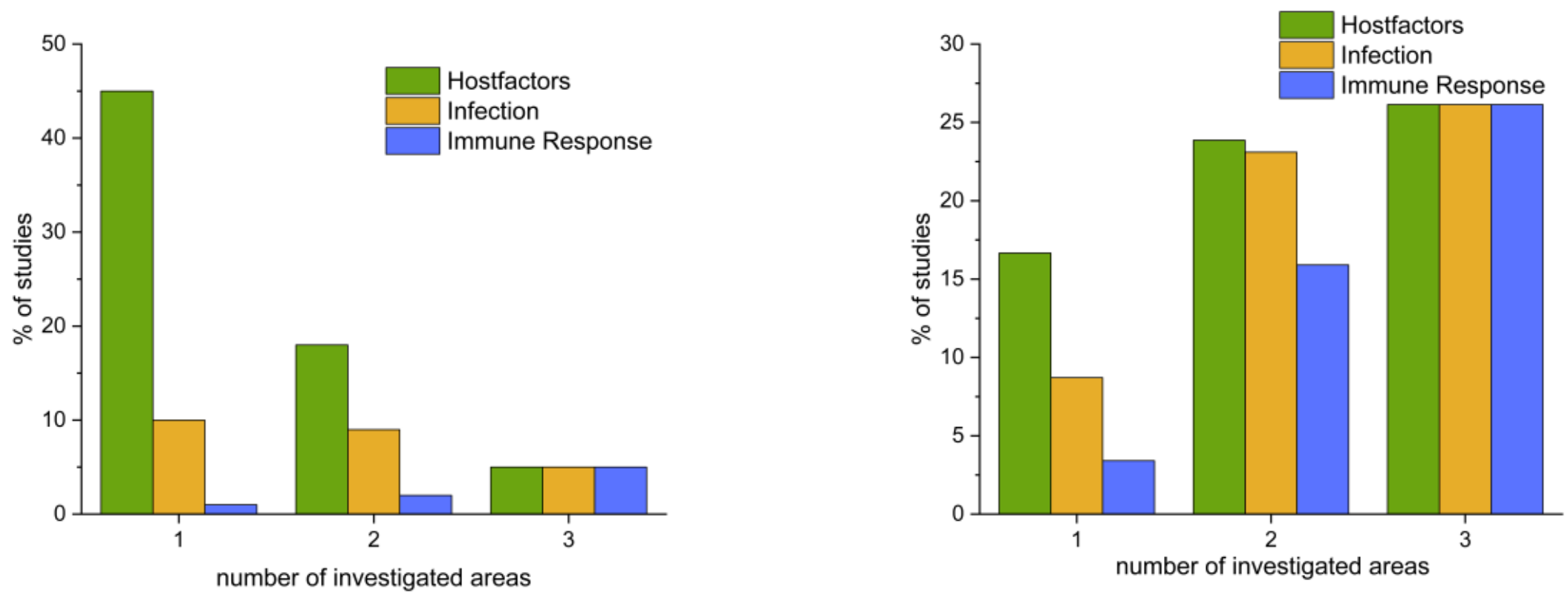

Supplementary Fig. 9: Number of investigated areas in the studies indicating how many studies (in %) focused on one, two, or three areas (host factors, infection, and immune response measurements)

Supplementary Figure 10: Forest plot based on posterior draw of ACE2 brms model (see also Supplementary Table 10)

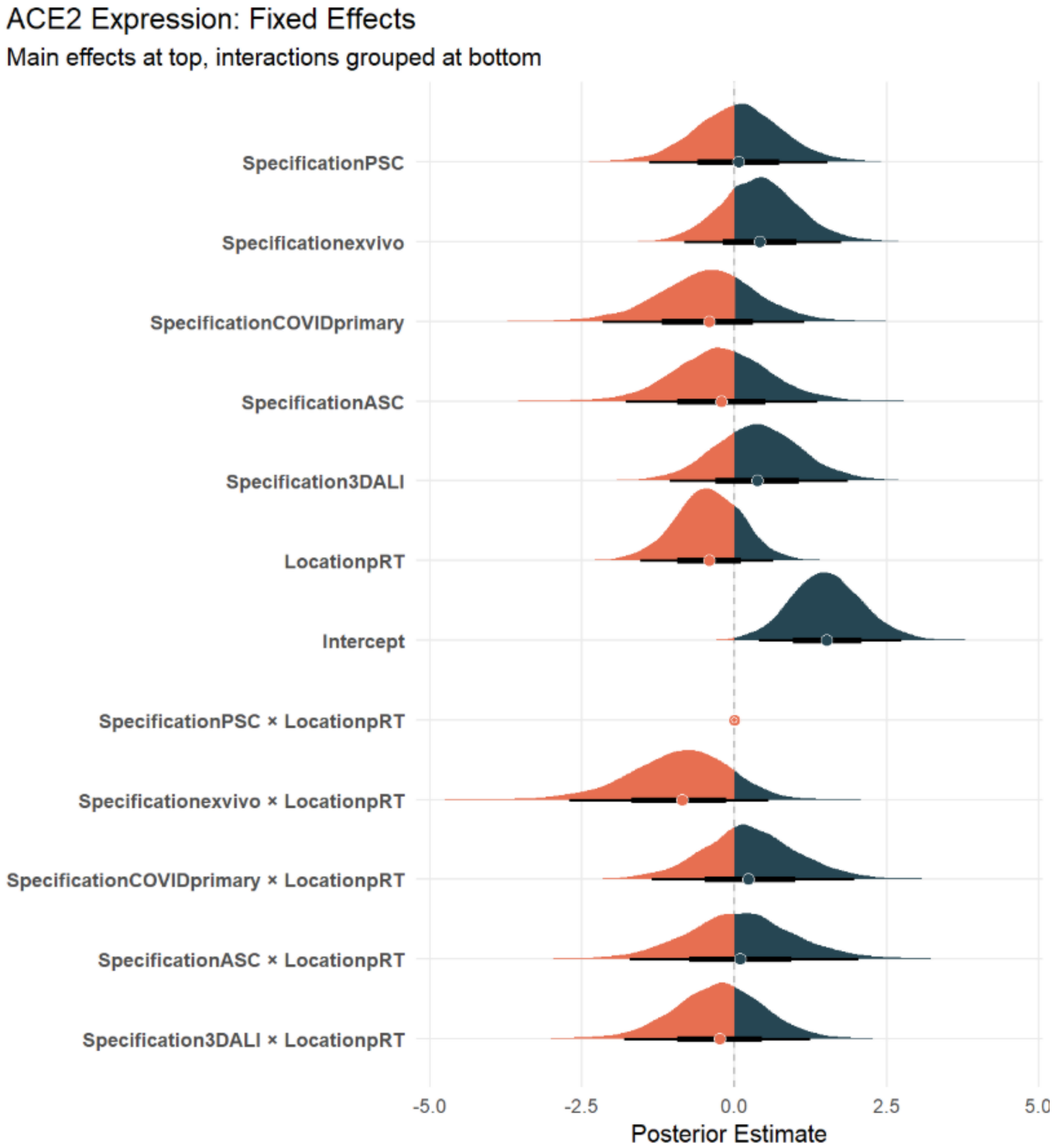

Supplementary Figure 11: Forest plot based on posterior draw of TMPRSS2 brms model (see also Supplementary Table 11)

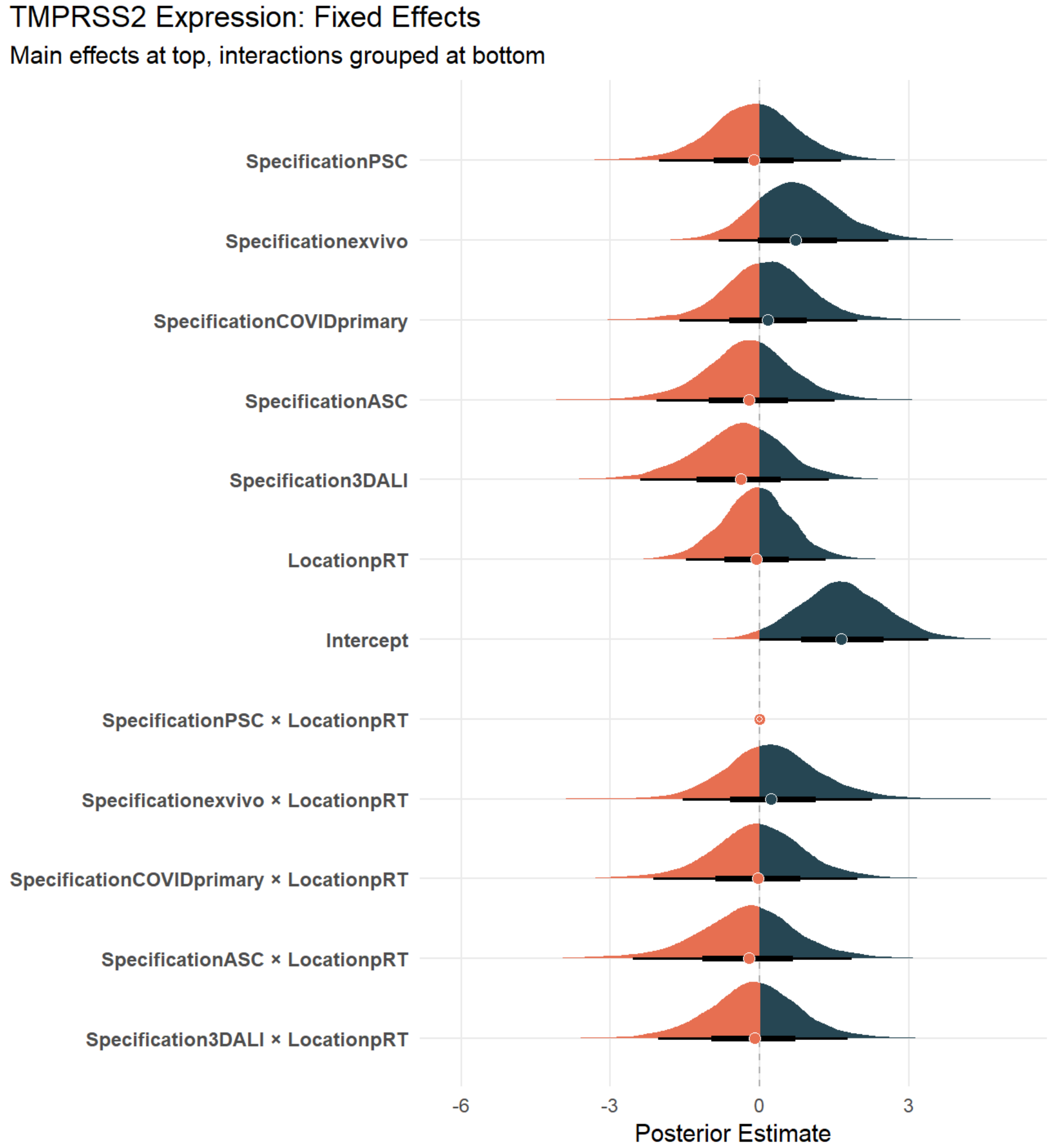

Reference list

(1) Zwietering, M. H.; Jongenburger, I.; Rombouts, F. M.; Van't Riet, K. Modeling of the bacterial growth curve. *Applied and environmental microbiology* **1990**, *56* (6), 1875-1881.
